# Devices Based on Co-Integrated MEMS Actuators and Optical Waveguide: A Review

**DOI:** 10.3390/mi7020018

**Published:** 2016-01-25

**Authors:** Franck Chollet

**Affiliations:** FEMTO-ST Institute, Université Bourgogne Franche-Comté, CNRS UMR 6174, 15B Avenue des Montboucons, 25030 Besançon cedex, France; franck.chollet@femto-st.fr; Tel.: +33-(0)3-6308-2622

**Keywords:** co-integration, MEMS, actuator, integrated optics, waveguide

## Abstract

The convergence of Micro Electro Mechanical Systems (MEMS) and optics was, at the end of the last century, a fertile ground for a new breed of technological and scientific achievements. The weightlessness of light has been identified very early as a key advantage for micro-actuator application, giving rise to optical free-space MEMS devices. In parallel to these developments, the past 20 years saw the emergence of a less pursued approach relying on guided optical wave, where, pushed by the similarities in fabrication process, researchers explored the possibilities offered by merging integrated optics and MEMS technology. The interest of using guided waves is well known (absence of diffraction, tight light confinement, small size, compatibility with fiber optics) but it was less clear how they could be harnessed with MEMS technology. Actually, it is possible to use MEMS actuators for modifying waveguide properties (length, direction, index of refraction) or for coupling light between waveguide, enabling many new devices for optical telecommunication, astronomy or sensing. With the recent expansion to nanophotonics and optomechanics, it seems that this field still holds a lot of promises.

## 1. Introduction

The birth of integrated optics, and more exactly the start of the technology enabling optical waveguide fabrication, may be traced back to the fine team of researchers at Bell’s Lab at the end of the 60s. In fact, the seminal work appeared in a special issue of the Bell System Technical Journal in 1969 [1], and proposed technologies and theories for fabricating and modeling waveguides, opening the path to what is now known as integrated optics.

It is rather eye opening to realize that the start of the MEMS technology, which enables fabrication of mechanical elements and actuators with microelectronics-like microfabrication techniques, dates back to the same era. In 1965 a team of researchers at Westinghouse research labs developed the resonant-gate transistor [2] that showed a remarkable integration of mechanical resonator with a field-effect transistor. Still, from this early work, the MEMS technology had to wait for the late 1980s to bloom, and actually the term MEMS itself was only coined in 1989 by Professor Howe at a notorious MEMS conference [3].

**Figure 1:**
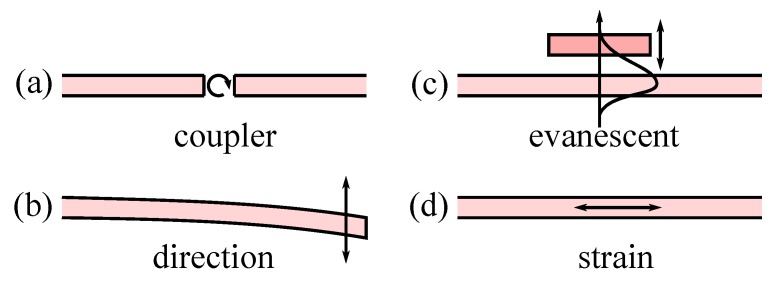
Principles of modification of light propagation in waveguides by mechanical actuation (**a**) coupling of fixed waveguide (**b**) redirection of waveguide (**c**) index of refraction change by interaction with evanescent field (**d**) change of waveguide geometry (strain).

It was only a matter of time before these two technologies would converge and at the beginning of the 1990s, several research groups in Asia [4,5], Europe [6,7] and America [8] proposed to use MEMS actuator with waveguide for a large range of different devices, from simpler sensors to optical telecommunication switches. Researchers have over the years explored different paths for building those devices where a MEMS actuator is able to modify the propagation of the light in a waveguide. Currently we can identify four simple principles schematically depicted in Figure 1. One of the first principle explored [5] (Figure 1a) was based on changing the coupling between fixed waveguides using a MEMS actuated mirror and, for example, could be used for optical switch fabrication. In this review, we will only consider the devices based on co-integration of waveguide and MEMS and will not discuss the other interesting devices based on optical fiber integration with MEMS actuator [9,10]. Similarly, directly changing the direction of a waveguide (Figure 1b) is a powerful method to channel the light between different waveguides. Then, it is possible to change the propagation of the wave in the waveguide, either by interacting with the optical field outside the waveguide (Figure 1c) or by directly modifying the refractive index of the material by inducing longitudinal strain (Figure 1d).

The question may arise, why in the first place would we want to use mechanical actuation whereas integrated optics has its own way of modifying light propagation in waveguides. Electro-optic, acousto-optic, and thermo-optic effects or the injection of free-carrier in semi-conductors are all very efficient principles for changing the index of refraction of materials, hence opening fabrication of high-speed or complex optical circuit for telecommunication or sensing. If they are fast—much faster than MEMS actuator—these principles, rooted in material properties, have very little magnitude (change of index of refraction in the 10^−3^ range) and in classical devices needs relatively long distance (cm) to obtain useful effects. Although photonic crystals devices using slow light [11] may be very small, a need for a compact integrated optics technology remains. It can definitely be achieved with MEMS actuators as the effect of moving optical elements is stronger than material effects. Moreover, the ability to act on the waveguide itself, changing its direction or length, opens-up new possibilities by allowing action principles previously impossible, potentially providing a new paradigm for photonics.

From the early days, an important issue of this new technology remains the co-integration of optical waveguide and MEMS actuator [12]. Simple sensing devices, where the actuation energy actually comes from the environment (vibration, pressure, acceleration, *etc.*), were from the beginning fully integrated [4] but it proved more difficult when the was actuator powered internally. In fact, among the first devices developed most used crude integration [5,7,8] and only the group of Voges at TU Dortmund in Germany [6] proposed a complete co-integration process. However, in our view, the co-integration is an important enabling technology that would allow building of array of devices (e.g., switch or sensor array), facilitate batch fabrication, limiting assembly and ultimately lower the cost.

Actually we will start our review of this particular class of optical MEMS [13] by exploring the different co-integration strategies that have been proposed since the emergence of the MEMS/waveguide technology. Then, we will describe the most important devices that have been based on this technology classified by the operating principles shown in Figure 1.

## 2. Fabrication Processes for Co-integration of Optical Waveguides and MEMS

### 2.1. Waveguide Technology

**Figure 2:**
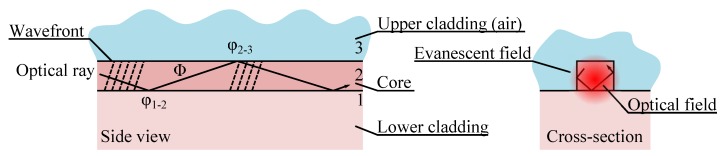
Side and cross-section view of light propagation in a waveguide (the cross-section view shows superimposed the optical field).

Guiding optical wave over long distance is commonly based on total internal reflection (TIR) where light in a high index material bounces on the interfaces with lower index materials. Figure 2 shows schematically the propagation of rays along a waveguide, with the corresponding wavefront. Basically, for avoiding destructive interference (between rays 1 and 3) that would spoil the optical wave, the accumulated phase shift from the reflection at both interfaces and the propagation across the waveguide needs to be a multiple of 2*π*, that is: 2Φ + *φ*_1−2_ + *φ*_2−3_ = 2*kπ*. Verifying this condition means that only a limited set of angles *θ* is possible, each of them corresponding to a mode of propagation of the waveguide with a specific velocity and optical field distribution. In this respect waveguides may be split between single-mode and multimode, depending whether they support a single or multiple modes at the wavelength of interest. In general for sensing or telecommunication applications single-mode waveguides are preferred because they exhibit low dispersion, while multimode waveguides are used for illumination applications. The simplest method for controlling the number of modes supported by a certain waveguide is to decrease its dimension, with smaller waveguide supporting less and less propagation modes. Figure 2 cross-section shows the fundamental electromagnetic propagation mode in the waveguide that can be derived from a wave analysis of the mode. Such analysis will also allow to take into consideration the light polarization that would basically show two types of mode in rectangular waveguide, the TE mode (with electrical field parallel to the substrate) and the TM modes (with magnetic field parallel to the substrate). The complete mode picture is actually more complex [14], but it will be sufficient for the discussion in this review.

By looking at the fundamental mode we notice that the optical field extends outside the core of the waveguide as an evanescent tails that decays exponentially. Basically, this tails extend deeper into the cladding medium if the relative difference of index of refraction (or index contrast) is low. If the optical field tail extends in air, it will allow interaction with another structure, modifying the propagation of the light, which correspond to the operation principle (c) in Figure 1. Increasing the evanescent field may be obtained by designing a waveguide close to the mode cut-off wavelength.

Moreover, this evanescent tail shows that the optical field “sees” the interface between the materials, and all the unevenness that may be present there due to the fabrication process. It has been shown that the higher the index contrast is, the more loss would be induced by scattering on the interface asperities [14]. The fabrication process of optical fiber (pulling of fiber and thick cladding) is very different from the techniques used for integrated optics, which are derived from techniques used in microelectronics (photolithography, etching of thin-films/cladding). Then the scattering effect due to the interfaces roughness is much larger than in optical fiber and the typical propagation loss goes from 1 dB/km to 1 dB/cm. However we should realize that the compactness of most integrated optics devices (length in the order of cm or less) makes the loss figure of the waveguide much less important than for optical fiber, and even high-loss technologies remain useful.

The significance of the refractive index contrast makes it an interesting yardstick to evaluate a certain waveguide technology and we list in Table 1 the main features of two typical waveguide technologies with high and low refractive index contrast. It should be noted that waveguide may have different contrast along the vertical and the in-plane directions, and in such a case, more complete analysis is required, although the general guidelines given in the table still hold.

**Table 1:** Features of index guiding waveguides with high or low refractive index contrast (*λ* refers to the wavelength of the guided light).

Properties	High refractive index contrast	Low refractive index contrast
Radius of curvature	short	large
Propagation loss	high	low
Mode field	small	large
Coupling with optical fiber	bad	good
Evanescent field in air	large	small
Waveguide width	*λ* and sub-*λ*	several *λ*

**Figure 3:**
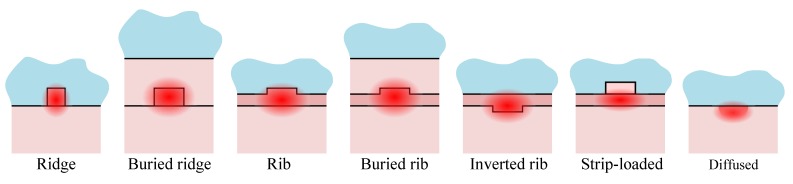
Cross-section of different types of waveguide based on TIR superimposed with their fundamental mode optical field (darker material have a higher index of refraction).

If the vertical refractive index contrast is usually obtained by the deposition of thin-films of different index of refraction, the in-plane refractive index contrast that defines the channel waveguide is obtained by patterning of the stacked layers. The most common architectures are shown in Figure 3, where the darker materials have higher index of refraction.

The rib and inverted rib waveguides do not employ materials of different index for lateral confinement and largely rely on a change in effective index arising from the structure, resulting in high simplicity in fabrication only surpassed by the ridge waveguide. The edge roughness of the etched structures induces scattering loss and the rib waveguide is worse in this aspect while most designs try to keep the mode profile buried below the surface and its deleterious scattering effect.

For keeping single-mode operation, the width of the patterned channel should be small and depends heavily on the index of refraction of the material and on the index contrast. Actually these two factors directly contribute to the phase shift during the wave zigzag (see Figure 2), the index affecting the phase accumulated while traveling (Φ) and the contrast governing the phase shift at the interface (*φ_i−j_*). For example if the index of refraction of the material is high (e.g., for Si *n* = 3.5) and the contrast large (e.g., Si/Air) single-mode ridge waveguide at *λ* = 1.5 μm will require a core with width in the order of 300 nm. In the other hand for a low-index-low-contrast waveguide, as found in an optical fiber, the core could be in the order of 9μm. The rib, the inverted rib, the strip-loaded or the diffused channels may yield a small in-plane index contrast, helping to relax the patterning dimension constraint.

Besides optical wave transmission using TIR there are other means for guiding light. Actually, as propagation loss in the 1 dB/cm range or even larger is acceptable if the device waveguide is short enough, even intrinsically lossy guiding principles may be implemented. The simplest is to use a hollow core and use fully reflective walls that will also create a pseudo-TIR. This principle has been used with success for microwave and also for integrated optics, either with metallic mirror walls, photonic crystal (PhC) arrangement [11] (generally showing in-plane only photonic crystal guiding while classical TIR is used in the vertical direction) or multilayer reflective dielectric coating (ARROW waveguide [15]). The ARROW waveguide is actually based on a core with a lower index (e.g., SiO_2_) surrounded by high index multilayer reflective coating (e.g., Si_3_N_4_/SiO_2_). Finally yet another possibility is to use plasmon resonance in metal strip, but in that case the device has to be very short (a few μm) because the loss becomes very large.

We have compiled in Table 2 the different waveguide technologies used in the literature with the working principle of the devices. The vast diversity of technologies used is a clear indicator that there is not a single Swiss’ knife waveguide process that would work in all cases. Moreover, if the device operation principle and the integration issue are important factors of choice, it is clear that another contributor lies in the prior existence of a process in the lab/company.

**Table 2:** Comparison of waveguide technology from literature (materials of the lower cladding/core/upper cladding, channel structure from Figure 3, principle from Figure 1).

Materials	Channel	Principle	References
Air/Si/Air	ridge	evanescent	[16,17,18,19,20,21,22,23,24,25,26,27,28,29]
		coupler	[30]
		direction	[31]
	PhC	evanescent	[32]
	slot	evanescent	[33]
GaAs/AlGaAs	ridge	direction	[34]
glass/doped glass/Air	diffused	evanescent	[8]
InP/InGaAsP	ridge	direction	[35,36]
		evanescent	[37,38,39,40,41,42]
metal/Air/metal (hollow)	reflection	evanescent	[43]
polymer/Air	ridge	coupler	[44,45,46,47]
polymer/polymer	ridge	direction	[48]
	buried ridge	coupler	[49]
SiO_2_/glass/Air	rib	strain	[4]
SiO_2_/Au	plasmon	evanescent	[50]
SiO_2_/Al_2_O_3_/SiO_2_	ridge	direction	[51]
SiO_2_/BK7/SiO_2_	strip-loaded	strain	[52]
SiO_2_/PS/Air	inverted rib	strain	[53]
SiO_2_/PSG/Air	rib	evanescent	[54]
SiO_2_/PSG/SiO_2_	buried rib	direction	[55,56,57,58,59,60,61,62]
		coupler	[63,64,65]
	buried ridge	coupler	[28]
SiO_2_/Si/Air	rib	coupler	[26,27]
SiO_2_/SiON/Air	rib	evanescent	[66,67,68,69]
	rib	strain	[70]
	rib ARROW	strain	[71]
	ridge	evanescent	[72,73,74,75]
SiO_2_/SiON/SiO_2_	buried ridge	coupler	[76]
	strip loaded	strain	[77,78,79]
	rib	evanescent	[80]
	inverse rib	direction	[6]
	buried rib	direction	[81]
SiO_2_/TiO_2_/Air	planar	evanescent	[7,82,83,84]

Quite naturally we see that devices based on evanescent interaction use air as upper cladding, creating large refractive index contrast in the vertical direction. We also notice that channel patterning is often done using low refractive index contrast structure (rib, diffused), helping somewhat decrease the constraint on dimension so that standard contact UV photolithography (critical width above ≈ 1.5 μm) may still be used. A notable exception to this rule is the Air/Si/Air platform with ridge waveguide, a high refractive index contrast technology, requiring ebeam or DUV lithography for patterning the sub-μm channel and advanced etching techniques for reducing sidewall roughness (e.g., Fast Atom Beam [19]) to keep the propagation loss below 1 dB/cm. Actually the main drawback of this technology—when the patterning issue is solved—is the poor coupling performance with optical fiber, but this can also be controlled with short taper structures (about 200 μm long) that decrease optical fiber coupling loss below 1 dB [85,86].

The main low refractive index contrast technology is based on phosphorus doped glass (PSG) and has been used by different teams for producing devices based on coupling or direction change exhibiting very low propagation (0.1 dB/cm) or fiber coupling loss (<0.5 dB). However, because of the relatively large dimension of the waveguide, this technology is unsuitable for devices based on evanescent or strain principles. A few groups have tried to push the co-integration further, by using active materials like InP or AsGa. In this case it is possible to envision devices where next to the passive waveguide and the MEMS actuator we build active optoelectronic sources and detectors—but the trade-offs could be too hard to master.

### 2.2. MEMS Actuator and Waveguide Co-integration

Since the onset of MEMS technology the issue of integration with other technologies has been a field of intense research. The integration of electronic circuits with MEMS structure for building smart sensors has spurred a lot of efforts, eventually resulting in monolithic integration with the iMEMS© process at Analog Devices [87]. At the same time an equally successful strategy has been to follow an hybrid integration approach with electronics and MEMS chips in the same package, using either flip-chip or side by side chips in a more classical System in Package (SiP) approach.

The co-integration of MEMS actuator and waveguide has followed the same path, with demonstrated monolithic, stacked and hybrid approaches.

The advantages of monolithic integration are well known (reduced size, reduced parasitic, direct path to array fabrication) but it carries its share of drawback and tradeoff because the technology caters both for waveguide and MEMS actuator. For avoiding a substantial increase in the number of process steps and a correlated decrease in fabrication yield, it is possible to use the same material for the waveguide and the actuator. In this case, the tradeoff is particularly severe, as the material must satisfy simultaneously optical requirements (optical index, layer thickness) and mechanical requirements (stiffness, mechanical stresses).

**Figure 4:**
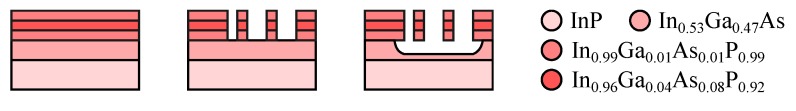
Example of monolithic integration process based on InGaAsP ridge waveguide and actuator (adapted from [36,39]).

In the example in Figure 4, the InGaAsP layers serves as structural and optical layer while the In_0.53_Ga_0.47_As layer serves as sacrificial layer as it may be easily dissolved with a mixture of HF and hydrogen peroxide, without affecting the phosphorus-containing layers. We note that the narrow structures etched in the waveguide layer stack becomes free while the broad structures remain anchored to the substrate, allowing to obtain the desired moveable and fixed structure. The process looks deceivingly simple, however it shows the kind of tradeoff required in monolithic integration: the InGaAsP layers are doped to make them conductive and allow powering the electrostatic actuator, but, if the doping is too high, they will start absorbing light through free carrier absorption, and the optical loss in the waveguide will increase dramatically.

A simpler path to monolithic integration is offered by stacked integration. This strategy requires less material tradeoff by stacking multiple layers with some layers geared toward actuator fabrication and others toward waveguide fabrication—but in that case the fabrication process becomes much more involved and presumably more costly. Other issues with stacked integration may impact the device operation and fabrication: for example, stacking will normally place the actuator and the waveguide in different planes, making their interaction more challenging, and the process will require a planarization step if the topography of the patterned lower layer is not to deform the top layer.

**Figure 5:**
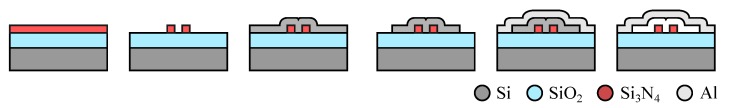
Example of stacked integration process based on a SiON ridge waveguide and a metal actuator (adapted from [75]).

We present in Figure 5 a typical stacked integration where an electrostatic actuator is placed over a pair of ridge waveguide in Si_3_N_4_ in order to interact with the evanescent optical field and spoil the coupling between the waveguides. In the stacked integration, we see that we may use different materials for the actuator and the waveguide. Still these materials need to be compatible (patterning the upper material should not destroy the lower material), and will often require a buffer sacrificial layer (here polysilicon deposited and patterned in the third and fourth steps respectively) that separates the waveguide from the actuator and is normally dissolved in the last process step. We note here that no planarization steps of the buffer layer has been introduced (after the third step), and it is obvious that the bumps and valleys of the patterned waveguide layer change the topography of the Al layer placed on top.

The hybrid approach relies on fabricating MEMS actuator and waveguide separately. Its main advantage is the possibility to use the best technologies both for the waveguide and for the MEMS actuator without compromise before they are assembled together. At the difference of electronics co-integration, most hybrid waveguide/actuator co-integration strategies are based on wafer level assembly—with appropriate bonding technology—and avoid device level assembly (e.g., flip-chip or SiP). The main difficulty of hybrid integration rests in the bonding step. Actually, besides presenting the difficulty of developing a reliable bonding technique, hybrid integration becomes complicated if precise alignment is required between the two wafers.

**Figure 6:**
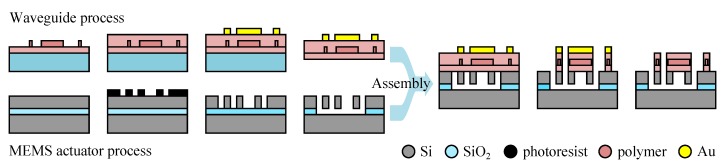
Example of hybrid integration process based on polymer buried waveguide and silicon actuator processes (adapted from [49]).

We show in Figure 6 a typical example of an hybrid fabrication process. The waveguide is a buried ridge type made of 3 layers of polymer with a patterned core on a glass substrate. We note here that the last step, that would define the lateral cladding after the gold mask deposition, is left for after the assembly, so that the waveguide layer is continuous and much simpler to manipulate and assemble with the actuator wafer. This second wafer is fabricated using typical SOI process [9], and we notice that the last step before the assembly is the release of the silicon structure. This step is more commonly found after the assembly as the unreleased structure is stiffer facilitating the relatively harsh assembly process. However in the work presented in Figure 6 the assembly step uses capillary force, making this order of process steps appropriate.

We attempted to show an exhaustive panorama of the strategies adopted in the literature for MEMS actuator fabrication and co-integration in Table 3. From the actuation principle point of view, we see an overwhelming majority of actuators based on electrostatics, either using comb-drive actuators or gap-closing actuators [88]. Only a few other principles are applied like thermal or magnetic actuators. Somewhat surprisingly, the table reveals that monolithic co-integration is favored over hybrid co-integration, although we see that the stacked integration is often preferred to a true monolithic process where the same layer is used for actuator and waveguide.

**Table 3:** Comparison of actuator technology and co-integration from literature (materials of the actuator, principle of operation of the actuator, co-integration type).

Materials	Principle	Co-integration	References
Au	gap-closing	monolithic	[50]
fluid	thermal	hybrid	[63,64]
	electrostatic	hybrid	[65]
GaAs/AlGaAs	comb-drive	monolithic	[34]
glass	(sensing)	hybrid	[7,82]
InGaAsP	gap-closing	monolithic	[35,36,39]
	light	monolithic	[40]
	(sensing)	monolithic	[41]
InP	gap-closing	monolithic	[37]
metal	gap-closing	hybrid	[5,54]
		stacked	[72,74,75]
Ni/Fe	magnetic	hybrid	[55,56]
polymer/metal	gap-closing	monolithic	[8]
poly-Si	gap-closing	hybrid	[28]
Si	gap-closing	hybrid	[67,68,69,73,89]
		monolithic	[20,21]
	(sensing)	monolithic	[30]
		stacked	[4,52,53,70,71,77,78,79]
SiON	gap-closing	hybrid	[66,83]
		monolithic	[57,58,61,62]
	(sensing)	hybrid	[84]
		monolithic	[6,51,59,60,81]
SOI	comb-drive	hybrid	[49]
		monolithic	[19,22,24,25,26,27,31,32,48]
		stacked	[76]
	gap-closing	monolithic	[16,17,23,29,43]
	(sensing)	monolithic	[33]

If the recent widespread use of the SOI process, yielding ridge channel with Air/Si/Air structure, shows the sign of a convergence toward a universal platform with many strengths, the large range of processes that have been developed over the years clearly shows that there is no simple answer to waveguide and MEMS co-integration. A proper choice of the right platform will have to be based on consideration about the device operation: overall loss in the device (*i.e.*, coupling and propagation loss), strength of the evanescent field in air, type of actuator, monolithic/stacked/hybrid integration, compactness...

## 3. Devices Based on Coupling Between Fixed Waveguides

### 3.1. Design Consideration

A direct integration of bulk optical circuits results quite naturally in the use of fixed waveguide that are coupled by an external element, the coupler (Figure 1a). From the literature we see a large diversity of movable couplers, be a simple mirror, a fluid, a network of waveguide or a Bragg reflector.

Those devices have to face a particular challenge as, paradoxically for a waveguide based device, they include propagation in free space. This strongly influences the loss of the device as it now faces two new sources of optical loss: beam spread due to diffraction in open space and Fresnel reflection at each interfaces.

**Figure 7:**
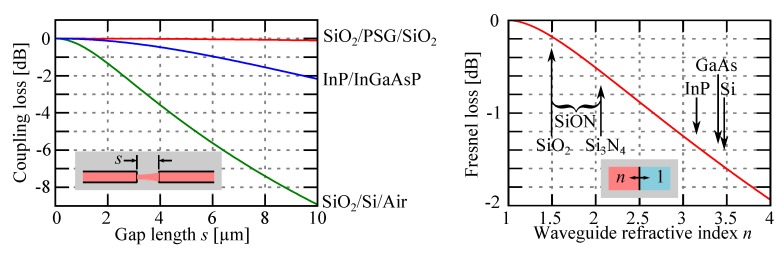
(**Left**) Diffraction loss (*λ* = 1550 nm) between two butt-coupled fixed waveguides of different technologies (SiO_2_/PSG/SiO_2_ [65], InP/InGaAsP [36], SiO_2_/Si/Air [27]), as a function of the separation distance *s*. (**Right**) Reflection (Fresnel) loss at an interface between a material of refractive index *n* and air.

Diffraction effect will steadily enlarge the beam propagating in free space and ultimately lower the coupling with the receiving waveguide. A complete analysis will require to know the exact mode profile of the waveguide but some insight may be gained by looking at the expression from Gaussian beam coupling, although approximating the waveguide mode field by a Gaussian is only somewhat valid for low refractive index contrast waveguide. Assuming an elliptical Gaussian mode, the power fraction *γ* coupled from one waveguide into another waveguide after propagation of a distance *s* is given by:

(1) $$\gamma \left(s\right)=\frac{1}{\sqrt{1+\lambda^{2}s^{2}/4\pi^{2}w_{0x}^{4}}}\frac{1}{\sqrt{1+\lambda^{2}s^{2}/4\pi^{2}w_{0y}^{4}}}$$

where *w*_0*x*_ and *w*_0*y*_ are the beam width in the X- and Y-direction, respectively, and *λ* is the wavelength of light in vacuum. Typical loss figure for some popular waveguide structures is shown in Figure 7 showing in general that the coupling element need to be relatively short for keeping diffraction loss below 1 dB. The coupling efficiency is also affected by other effects like the tilt or the offset of the waveguides that will lower even more rapidly the coupled wave [90].

Moreover when the light has to cross the interface between the waveguide and air, it is affected by Fresnel reflection, that will in turn lower the transmitted light fraction *T*:

$$T=1-R=1-{\left(\frac{n_{1}-n_{2}}{n_{1}+n_{2}}\right)}^{2}$$

where *n*_1_ is the refractive index of the core and *n*_2_ = 1 the refractive index of air. As can be seen in Figure 7, when the core material is SiO_2_ (*n* ≈ 1.5) we find a reflection of about 4% (0.17 dB loss/interface), but with Si (*n* ≈ 3.4) it reaches almost 30% (1.5 dB/interface) and will require to be decreased by using special coating or interference for obtaining useful devices [36]. As such, it makes sense that most devices based on external coupler are based on the SiO_2_/PSG/SiO_2_ waveguide technology.

### 3.2. Principle Application

**Figure 8:**
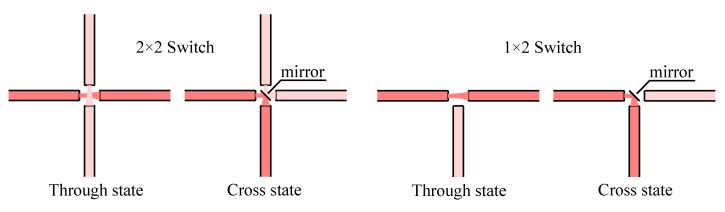
Principle of 1 × 2 and 2 × 2 optical switch [5,89] using a sliding flat mirror as coupler between fixed waveguides.

Historically, the first device using this technology was simply based on a metallic mirror that could be inserted at 45° between two perpendicular waveguides (Figure 8). In this case we obtain a 2 × 2 optical switch, with a “through state” where the light goes straight in the waveguide and the “cross state” where the inserted mirror diverts the light to the other waveguide [5]. We have also represented in the figure the influence of the gap between the two waveguides on the optical loss. We observe that if we use low refractive index contrast waveguides (e.g., SiO_2_/PSG/SiO_2_), the mode size becomes relatively large and the diffraction loss is kept very low. For high index contrast waveguide the figure is much worse and in that case the gap has to be kept very small for avoiding excessive loss - and of course Fresnel loss will also become higher. The diffraction loss is not the only loss that will affect this kind of mirror switch, as the roughness of the mirror has to be kept well below the wavelength (a rule of thumb is to keep Ra < *λ*/20 to keep loss at a few %) for avoiding scattering at the reflection. Accordingly, some investigators [89] have focused on producing good vertical mirrors using deep RIE silicon etching or high aspect ratio polymer patterning (*i.e.*, SU8 [91]). The author have measured a roughness of Ra = 21 nm on the silicon mirrors etched in an optimized Bosch process, well below the rule for operation at 1550 nm (*λ*/20 ≈ 75 nm), resulting in scattering loss of about 2.5% at an incident angle of 45°.

This switch may also be operated as a 1 × 2 switch when the light is reflected only on the front of the mirror. This is a more reliable configuration as the 2 × 2 version requires a very thin two sides mirror. Actually the thickness of the mirror will slightly offset the light beam upon reflection, increasing the loss of the switch [5]. In this case to avoid this issue it seems possible to use a transparent material for the mirror [89] and coat the thin-film reflecting layer on a single side. In any case, the interest of a 2 × 2 switch over a 1 × 2 switch becomes evident when the switches are combined to form *n* × *n* switch matrix, the former allowing for certain matrix architectures to divide the number of switch cells by a factor of 2.

**Figure 9:**
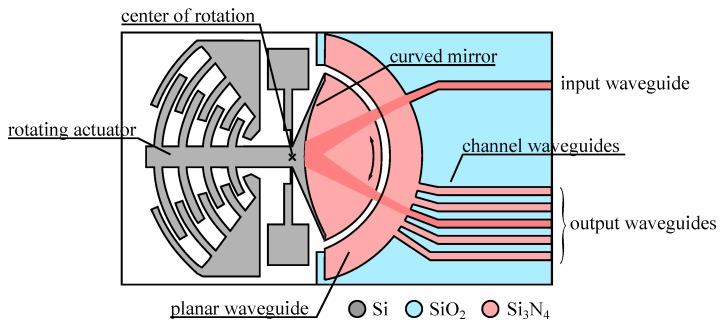
Sketch of a 1 × 5 optical switch [76] using a rotating mirror.

More recently, another co-integrated architecture has been proposed where the mirror is rotating for providing an 1 × *n* optical switch (Figure 9). Interestingly in this device [76] the length of free-space propagation (and the associated diffraction loss) is kept to a minimum by using a planar waveguide that will show diffraction in the in-plane direction only. This spread is then compensated by focusing the light with a curved mirror instead of a flat mirror. The complexity of the integration is mitigated by using a stacked approach and building all the optical elements (including the curved mirror) with the optical layers. Still, the stringent requirements for the rotation axis require a complex design based on hinges, that will need more refinement before a fully functional device is produced.

For deflecting the beam in the previous devices, we require a rather large stroke for the actuator, making it hard for the speed of these system to increase significantly. It is actually possible to take benefits of interference phenomenon for drastically reducing the actuator stroke to a fraction of the wavelength.

**Figure 10:**
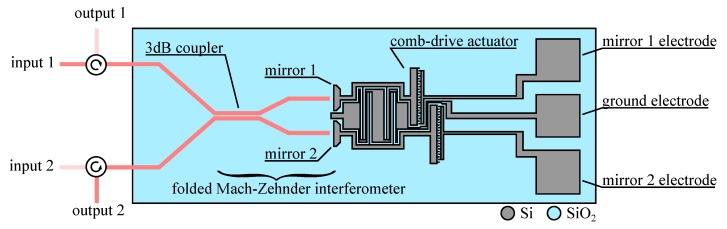
Sketch of a 2 × 2 optical switch based on a tunable folded Mach-Zehnder interferometer [26,27].

A group at Bell’s lab has proposed to use phase control in a folded Mach-Zehnder interferometer (MZI) for switching (Figure 10). The mirrors placed at the output of the two waveguides allow for controlling the length of each arm of the MZI. When the arm length are the same, the light that comes into the device at the top port will leave from the bottom port (and reciprocally). If one of the mirror is moved, it creates a phase difference *δφ* between the two arms and the output light will split continuously between the top and bottom port as sin^2^(*δφ*/2) and cos^2^(*δφ*/2) respectively. The circulators on the left allows for separating the incoming and outgoing light, effectively giving 2 input and 2 output ports in this folded configuration. A phase shift *δφ* = *π*/2 enables switching of the light between the two output ports, and since the light is folded at the mirror it is sufficient to move the mirror by *λ*/4. In this design this was obtained by applying 45 V on the comb-drive actuator and the switching speed reaches 150 μs that is about an order of magnitude faster than the other mirror based switches. The ability to adjust both mirror positions improves tremendously the manufacturing tolerance for reaching the initial state at *δφ* = 0 + 2*kπ*.

As the device is fabricated with monolithic co-integration, the waveguide and the actuator is also made of silicon on a SOI process. For reducing the Fresnel loss, the waveguide termination facing the mirror is antireflection (AR) coated at a 45° angle using a shadow mask, but the complete device still exhibits large loss (>10 dB). The possibility offered by integration of an integrated optics circuit and an array of mirror for controlling the phase of the reflected light, has been used by the same team for proposing a wavelength switch with 16 channels [28]. The device uses a similar principle as the Mach-Zehnder switch, except that an integrated planar circuit first splits spatially the 16 wavelength using an arrayed waveguide grating before the light is sent to the mirror. Three orders of diffraction of the grating are collected for improving the extinction ratio with an arrayed waveguide lens placed between the grating and the mirrors. The device is based on hybrid co-integration with low loss waveguide in SiO_2_/PSG/SiO_2_ for the complex planar lightwave circuit and an array of out-of-plane polysilicon mirrors built with surface micromachining.

The application of mirrors does not stop here as a team at EPFL has demonstrated an original architecture resting on a flipping mirror co-integrated with a hollow waveguide [43]. The group proposed a Y-branch hollow waveguide, with the mirror flipping on the side at the branch akin to an “optical switchpoint”. The difficulty arises from the technology of the hollow waveguide requiring a complex process for coating the inside of the waveguide on its four sides. The best results obtained used a gold coating exhibiting propagation loss in the order of 1.8 dB/cm. If the device suffered from a low contrast between the two operating states, it had a very high speed, as the switching could be obtained in 10 μs that is 100 times faster than with most MEMS based optical switches.

**Figure 11:**
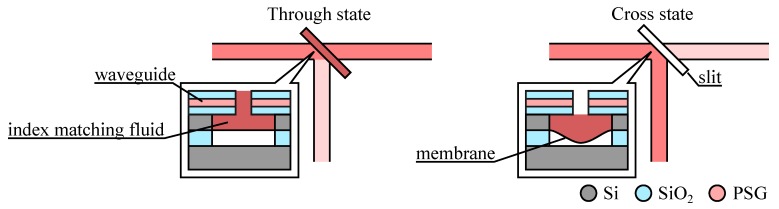
1 × 2 optical switch based on spoiling the total internal reflection with a fluid. (Insets) Cut-out view showing the electrostatic membrane actuator in the Through and Cross states (adapted from [65]).

Reflection is a powerful method for changing light direction, but it does not need to use an external mirror. It is in fact possible to use the total internal reflection appearing at a slanted waveguide/air interface and then to spoil this reflection by introducing an index matching fluid. As shown in Figure 11, when the index matching fluid is present at the interface, the light stops reflecting and goes straight. The built optical switches have relied on several principles for ensuring the motion of the fluid in and out of the slit. The first devices were based on thermally driven droplets (thermocapillarity effect [63] or vaporization [64]) using integrated heaters close to the slit, but one of their main drawback was that the heat would significantly change the fluid refractive index requiring special packaging scheme for mitigating this issue. The later device shown here [65], used an electrostatically driven membrane to push the fluid in and out of the slit, avoiding the problem of the thermal actuation.

This principle may easily lead to integration in large matrix, and up to 32 × 32 matrix [64] have been produced. However we notice that the overall loss was heavily dependent on the path (*i.e.*, the number of reflection), ranging between 2.6 dB and 6.9 dB in this later device.

**Figure 12:**
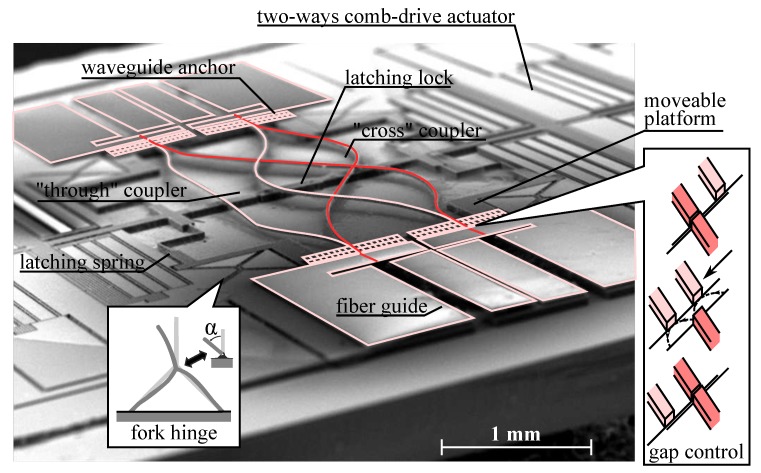
View of a 2 × 2 optical switch [49] using a network of waveguide on a moveable platform for coupling input and output. (**Inset Left**) Detail of the forked-hinge motion. (**Inset Right**) Arched motion responsible for the gap reduction mechanism.

Channeling the light between the fixed waveguides may be obtained not only by using mirrors but also by using a network of waveguides, in a manner similar to what was done in old manual switchboards. A 2 × 2 optical switch has been proposed where two networks of waveguide enables switch between the through and the cross state [49]. As we can see in Figure 12, a comb-drive actuated platform may place one of the two short stretch of waveguides configured as “cross coupler” and “through coupler” in front of the input and output waveguides, obtaining the two desired states of the switch. This device tries to remedy several problems identified with previous designs. Firstly, although it works with electrostatic actuator it is actually bi-stable, being able to maintain the cross and through state without power. This ability is conferred after fabrication by applying a high voltage to the comb-drive actuator to engage a mechanical lock and store energy in the latch spring. Then, applying a lower voltage either to the left or the right actuator will enable switching between the two stable states of the device. Additionally it was noticed that the diffraction loss may strongly affect this device as it has two sections of free-space propagation on both sides of the moveable platform. This foreseeable problem was circumvented by using an arched motion of the butt-coupled waveguide as they come into contact with the fixed waveguide, effectively reducing the gap to 0 (Figure 12-inset right). To facilitate this complicated motion the authors choose a compliant waveguide technology based on polymer waveguide with low stiffness (Figure 6) and designed a new type of hinge, dubbed the “fork hinge” [92], which yields large rotation angle in a buckling-resistant design compatible with the latching mechanism (Figure 12-inset left). The fabrication of the device was based on hybrid co-integration of a polymer waveguide wafer (supporting optical fiber alignment patterns) with the actuator wafer etched in SOI using the single step “etch & release” process [93]. The device showed a switching speed of about 0.5 ms and the optical loss were around 2.5 dB including rather high propagation loss of 2.88 dB/cm that were attributed to problems in the material processing.

A simplified version of this device was used to form an optical signal amplitude modulator or an ON/OFF switch. The device uses one single input and output waveguides coupled by a short section of straight waveguide placed on a mobile platform [31]. When the comb-drive actuator is powered the coupler waveguide on the platform approaches the end of the two fixed waveguides and progressively channels the light from the input to the output. The authors have also tried to reduce the gap between the waveguides in the ON state by using 45° inclined waveguide ends that enables “contact” between the translating and the fixed waveguides ends, but they haven’t made any effort to reduce the Fresnel loss. The monolithic co-integration on SOI wafer is based on Air/Si/Air ridge waveguides 500 nm wide and 260 nm thick. This choice gives a very compact device with small actuator stroke resulting in a relatively high modulation bandwidth of about 100 kHz. The achieved extinction ratio between ON and OFF states is about 15 dB while the loss of the device in the ON state is about 6 dB (that is 3 dB per coupling corresponding essentially to the Fresnel loss), excluding the fiber coupling loss that will be relatively high because of the small mode size in this high refractive index contrast waveguide.

We notice that all the devices we have described so far were used for building optical telecommunication components, but there are other applications of co-integrated waveguide and MEMS actuator that may be developed and in particular for sensing. In a sensor, the actuator energy is not coming from the device (in general, a form of electricity) but from the external measurand. Accordingly, actuator will be powered by acceleration, pressure, stress... for measuring these physical quantities.

**Figure 13:**
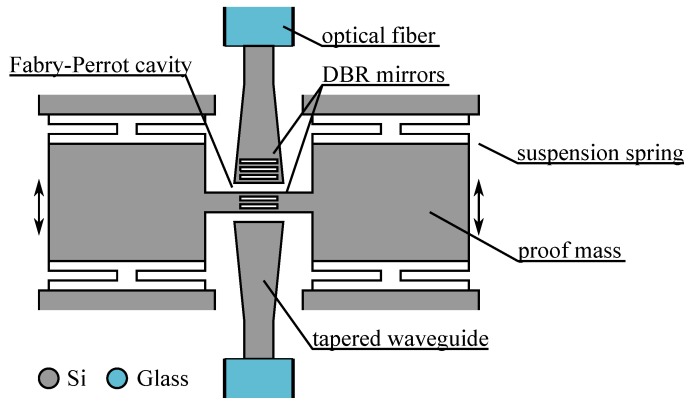
Acceleration sensor using Fabry-Perrot interferometer with a suspended DBR mirror coupled to tapered waveguides monolithically integrated on silicon [30].

For example, the measurand could change the transmission of a coupler placed between 2 butt-coupled waveguides. In one embodiment of this principle [30] the authors placed between the two waveguides a Fabry-Perot (FP) cavity (that is, two mirrors facing each other) with one mirror fixed and the other suspended. Acceleration imposed on the device will move the suspended DBR mirror, changing the FP-cavity length and shifting the transmission peak of the FP. Tracking the FP transmission peak will directly inform on the acceleration *a*, as the wavelength shift Δ*λ* is simply related to the suspended mirror resonant frequency *ω_n_* and the order of interference in the FP cavity *m*: $\Delta \lambda =-2a/m\omega_{n}^{2}$. The complete device could be co-integrated on a SOI wafer with a single etch as the mirror consisted of a distributed Bragg reflector with a series of 3 alternating air/Si slabs. The prototype sketched in Figure 13 had a cavity length of 27 μm resulting in an order *m* = 52 and a sensitivity of about 90 nm/g. The range of the accelerometer is limited by the free spectral range (FSR) of the FP (the transmission peak repeats itself every FSR) which is 0.26 g for this particular cavity, understanding that a shorter cavity will show a larger range and sensitivity. A similar device has also been fabricated on an InP/InGaAsP platform [40].

Finally, the capability of detection of light has also been explored in the so called Integrated Optical Metrology [44,45,46,47], where a network of waveguide is used to measure the position, deformation, rotation speed, *etc.*, of microstructures to get mostly positional feedback and enable closed loop control of MEMS. The hybrid principle of co-integration based on polymer waveguide has not seen a complete demonstration, but it is an interesting approach that may be useful in the future.

## 4. Devices Based on Modification of Waveguide Direction

### 4.1. Design Consideration

The possibility to change the direction of a waveguide (Figure 1b) is a very appealing mean of changing light propagation. The waveguide is then considered as a beam that is bent by applying a force with a MEMS actuator.

For describing this structure mechanically, the waveguide is seen as a cantilever with a force applied at a distance *L* from its anchor, that has a stiffness *k* given by

$$k=\frac{E}{4}\frac{hw^{3}}{L^{3}}$$

where *E* is the elasticity modulus of the cantilever material, *h* its thickness, *w* the width of the waveguide (in the direction of bending). For example an SiO_2_/SiON (*E* ≈ 70 GPa) cantilevered waveguide 2 mm long, 15 μm wide and 25 μm thick [62] will have a stiffness of *k* = 0.18 N/m, requiring a force of about *F* = *kδx* = 2 μN for moving its tip by *δx* ≈ 11 μm.

On the optical side, in addition to the Fresnel loss appearing at the exit facet of the waveguide, the bending may introduce additional losses due to the leaking field at the bent interface with increased reflection angle. The average radius of curvature $\bar{R}$ (*R* is decreasing linearly from $2\bar{R}$ at the anchor to 0 at the tip) in the cantilever is given by

$$\bar{R}=\frac{FL}{6Ehw^{3}}=3\frac{F}{kL^{2}}$$

and the attenuation coefficient is varying rapidly with the radius of curvature [94] as

$$\alpha =C_{1}e^{-C_{2}\bar{R}}$$

where *C*_1_, *C*_2_ are large constants depending on the straight waveguide geometry and the light wavelength. Then the attenuation *A* in dB in the bent waveguide may be estimated by *A* = 10log *e^−αL^* = −4.3*αL* and will thus degrade very rapidly with smaller $\bar{R}$. Still, for low refractive index contrast an average bending radius $\bar{R}$ above a few mm will create limited loss, whereas in high refractive index contrast, the radius may be below 100 μm and still yield acceptable loss figure. Actually for the silicon nanowire waveguides [85], the bending radius may be as small as 2 μm and still maintain loss below 0.5 dB.

We note also that if the light from the bent waveguide has to be coupled in another (fixed) waveguide, the device will also be affected by diffraction loss issue (Figure 8-right) as the light has to propagate in free space in the gap between the two waveguides and also by offset loss if the two waveguide ends are not perfectly aligned. The loss due to the waveguide tilt at the tip (tip slope is given by *θ* = *FL*^2^/2*EI* = 1.5*δx*/*L*) is normally very small compared to the other loss as for example *θ* = 0.2° for the 2 mm long cantilever above and *θ* = 2.3° for a 250 μm long cantilever with 10 μm deflection.

Accordingly, neglecting the tilt and Fresnel loss and using a Gaussian field approximation, the power coupling efficiency for a longitudinal separation *s* and a lateral offset *δx* is given by [81]:

$$\gamma \left(\delta x,s\right)=\frac{1}{\sqrt{1+\lambda^{2}s^{2}/4\pi^{2}w_{0x}^{4}}}\frac{1}{\sqrt{1+\lambda^{2}s^{2}/4\pi^{2}w_{0y}^{4}}}e^{-\frac{\delta x^{2}/w_{ox}^{2}}{1+\lambda^{2}s^{2}/4\pi^{2}w_{0x}^{4}}}$$

**Figure 14:**
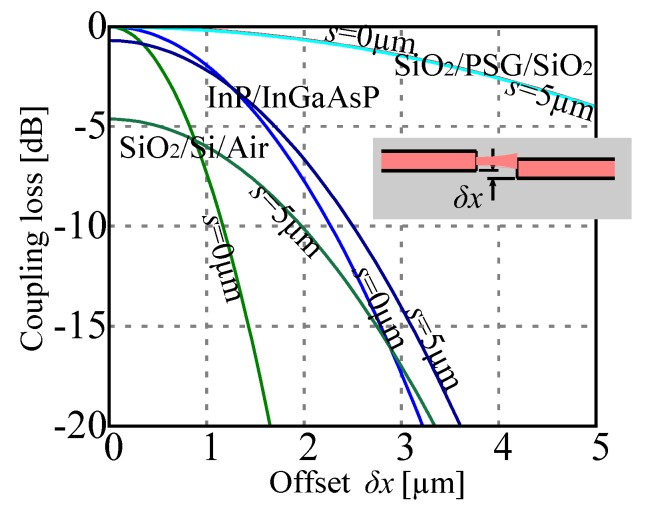
Coupling loss between two butt-coupled waveguides of different technologies (SiO_2_/PSG/SiO_2_ [65], InP/InGaAsP [36], SiO_2_/Si/Air [27]) as a function of lateral offset *δx* for two different gaps *S* = 0 μm and *S* = 5 μm.

This figure may rapidly reach several dB if the waveguide index contrast is high and the mode correspondingly small as we may see in Figure 14.

### 4.2. Principle Application

**Figure 15:**
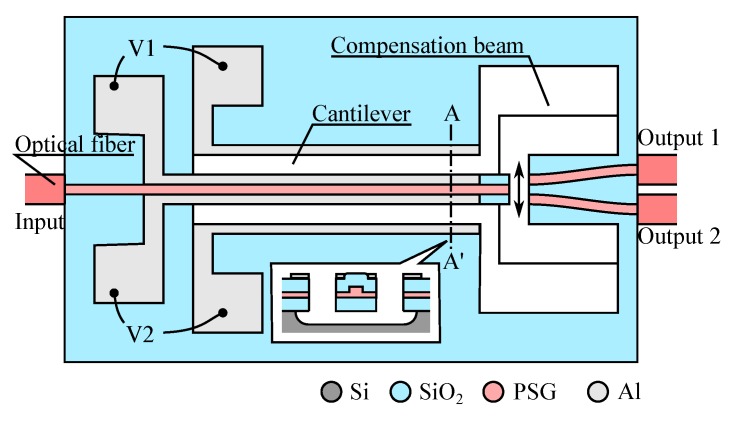
Example of 1 × 2 optical switch using control of direction of a cantilevered waveguide adapted from [62] (inset: cut-out view along A–A’).

One of the first idea that emerged from a team at LETI was to couple the bending waveguide with a pair of fixed waveguides and propose a 1 × 2 optical switch [57,58], as sketched in Figure 15-left. The application of a voltage *V*_1_ (respectively, *V*_2_) will move the cantilevered waveguide toward the output waveguide 1 (respectively, output waveguide 2) effectively channeling the input signal toward the desired output. The device is obtained by monolithic integration needing a trade-off between the actuator and the waveguide functions. In this early version of the device the electrodes placed on the waveguide sides form a gap-closing actuator but they were replaced by large comb-drives in later version [62]. This change allowed to separately optimize the geometry of the cantilevered waveguide and the actuator force. We note in this device the presence of “compensation beams” that were required to keep the cantilevered waveguide in the same plane than the two output waveguides. In fact, the stack of SiO_2_ layers in the waveguide (see inset in the Figure) is inevitably plagued by a stress gradient resulting in upward or downward bending, that is minimized by the presence of the compensation beams. The SiO_2_/PSG technology used for the waveguide has good fabrication tolerance as we may gather from the curves in Figure 15-right: even with an offset of 2 μm and a separation of 5 μm the loss remains below 1 dB. Accordingly, the switch shows nice characteristics with insertion loss in the order of 1.5 dB, and a behavior almost insensitive to the wavelength in a very wide range of wavelength (1150–1650 nm). This last figure is a major asset for switches that are not based on interference as it is usually the case in integrated optics technology, where bandwidth of 50nm are already considered wide. The speed of the switch is in the order of 1 ms, and even faster when there is no index matching fluid used in the gap between the waveguide (using the switch without gel increase the optical loss to 2.5dB). Multiple version of the switch have been produced by fully using the co-integration opportunity and cascading multiple 1 × 2 units, providing devices in 1 × 4 and 1 × 8 configuration [61,62].

This principle has seen many different versions trying to improve on some of this basic features.

A team at Hitachi proposed to use the cantilevered waveguide with an hybridized magnetic actuator for providing a dual 1 × 2 optical switch [55,56]. Using a combination of ferromagnetic sheet on the movable head and of permanent magnet and electromagnet on the stator they obtained a bi-stable actuator, able to maintain any of the two working positions without power consumption. The current flows in the electromagnet only when the switch has to flip from one output to the other. The insertion loss were high above 3 dB, presumably because the bi-stable nature of the actuator prevented continuous waveguide positioning that would have enabled to tune the waveguide coupling.

The original switch design used low refractive index contrast waveguide technology for ensuring low propagation loss and a good coupling with optical fiber, however this implied that the width and thickness of the waveguide had to be relatively large and consequently the cantilevered waveguide stiff. This characteristic results in a relatively large size of the switch for obtaining a softer cantilever (about 2 mm long) and/or integrate strong actuator. For reducing the waveguide dimension a simple path is to opt for a high refractive index contrast waveguide.

A research group at Sandia lab has proposed a 1 × 2 switch with monolithic co-integration using AsGa rib waveguide technology. They obtained a lower actuation voltage (down to 3.3 V for the 700 μm long cantilevered waveguide) and much smaller footprint (less than 500 μm long device for the shorter cantilever with actuation voltage below 12 V) [34]. Additionally, the stiffer structures showed a fast switching time below 50 μs that could still be improved by using excitation with overshoot.

The same team also proposed a cantilevered waveguide 1 × 2 switch based on an interesting stacked co-integration with SOI actuator and polymer waveguide placed on a Si cantilever resulting in a device with characteristics similar to the original device from LETI [48].

The possibility to integrate source or detector and use a high refractive index contrast waveguide technology attracted attention toward non-silicon technology. A group at the University of Maryland developed devices on the active indium phosphide (InP) technology, proposing a monolithic co-integration process for an 1 × 2 switch based on the same principle [35,36]. The propagation loss were a bit high at 2.2 dB/cm in the ridge cantilevered waveguide [38] but the compactness of the device ended with a total loss of 3.2 dB (excluding fiber coupling loss) and an actuation voltage of less than 7 V.

Active devices like switches were not the only devices proposed using waveguide direction change. When the actuator in Figure 15 is not powered, the suspended beam will be subject to acceleration and shift, changing the coupling behavior with a butt-coupled fixed waveguide as shown in Figure 14. The sensitivity to acceleration may be improved by increasing the weight attached to the cantilever, as the resulting force acting on it is given by *F* = *ma* where *m* is the mass of weight. The relative simplicity of the device (as integrating an actuator isn’t needed) made it one of the first application of the co-integration technology, with mostly monolithic devices developed in SiO_2_/SiON technology [6,51,59,60].

A slightly different approach used the beam as a resonator with eventually a measurand affecting its resonant frequency [81]. The resonator is put in vibration using a thermal actuator that took benefit of the bi-metal effect existing in the stack of layer used for the cantilever. By flowing AC current through a patterned metallic electrodes atop the cantilever, it could be excited up to 300 kHz. The vibration of the cantilever are measured by recording the coupling loss between the vibrating and the fixed waveguides. The author used a series of 8 cantilevers with varying resonant frequency that were integrated on the chip with a 1 × 8 fan-out waveguide network for their interrogation. Another possible use of this architecture could be in developing a frequency analyzer for sound, where the sound waves could put in vibration the series of cantilever with staged resonant frequency. Recent developments adapted this technique to the Si/Air nano-waveguide on SOI substrate for mass sensing [95]. Interestingly the need for good control of residual stress to obtain straight suspended waveguides was solved with Ar-plasma bombardment of the waveguide surface, a technique that could be applied more broadly for avoiding the use of tethering structures [62].

## 5. Devices Based on Interaction with the Waveguide Evanescent Field

### 5.1. Design Consideration

**Figure 16:**
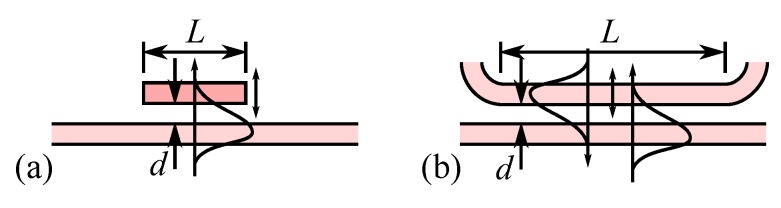
Evanescent interaction over a length *L* with a gap *d* between (**a**) a dielectric slab and a waveguide, changing the effective index of refraction of the mode (**b**) two waveguides, allowing exchange of energy.

The possibility to interact with the evanescent field of a propagating mode has, from an early period [96], been recognized as a powerful mean for switching light. Actually, there are two main ways to use this principle (Figure 16): either a slab of material is simply brought inside the evanescent field resulting in a modification of the effective refractive index of the waveguide mode, or, another waveguide is brought into the evanescent field opening the possibility to have an exchange of energy between the two waveguides.

In the first case (Figure 16a), a slab of material is dipped into the evanescent optical field using the MEMS actuator. If the material is close enough from the surface of the waveguide, the effect may be described by considering an effective index of refraction *N* seen by the mode of the waveguide. *N* = *n* + *ik* is complex, representing with the real part *n* the light velocity change and with the imaginary part *k* the light absorption, and depends on the slab material and on the distance between the slab and the surface of the waveguide. The magnitude of the index modulation decreases exponentially with the gap distance *d*, approximately as *exp*(−2*d*/Δ*z*) where $\Delta z=\left(\lambda /2\pi \right)\left(n^{2}-n_{air}^{2}\right)$ is the light penetration depth with *n_air_* the index of refraction of air [66]. In practice the maximum distance *d* where a significant effect still occurs is below *λ*/10, that is, generally below 100 nm, meaning that the actuator controlling this effect could have a short range—but requires high stability because of the high sensitivity (exponential variation) of the modulation effect with *d*.

If the slab is not absorbing at the wavelength of interest it induces a change of the real part of the effective index of refraction Δ*n* and we get phase modulation. This change over a zone of length *L* induces a modification in the phase of the mode field by an amount:

$$\Delta \phi =2\pi \frac{L}{\lambda }\Delta n$$

This phase shift may be in turn transformed into intensity modulation by using an interferometric scheme.

If the slab is made of a material absorbing light at the wavelength of interest, the change will affect the imaginary part of the index of refraction Δ*k* and light is absorbed giving direct intensity modulation. For a zone of length *L* the attenuation of the mode field is given by:

$$T=e^{4\pi \frac{L}{\lambda }\Delta k}$$

We note that in both cases we have different index changes for the TE and TM polarizations.

In the second application of this operating principle (Figure 16b), the two waveguides brought in close vicinity may both propagate light. In that case, in addition to the refractive index change seen previously, if the right condition are met, we observe coupling and exchange of energy between the two waveguides.

For understanding this evanescent coupling we may, in a first approximation, describe it with the coupled-mode theory in the weak coupling regime [73,97]. In this configuration a set of coupled equations relates the slowly varying amplitude, *a*(*z*) and *b*(*z*), of the two coupled modes propagating in the structure. Solving these equations for two waveguides separated by a low index gap and with relevant boundary conditions gives the irradiance of the two modes, *I_a_* and *I_b_*, with the propagation distance *z* as:

(2) $$\begin{array}{lll} I_{a}\left(z\right)=a\left(z\right)a^{*}\left(z\right) & = & I_{a}\left(0\right)\left[\cos^{2}\left(\sqrt{\kappa^{2}+\delta^{2}}z\right)\right.+\left.\frac{\delta^{2}}{\kappa^{2}+\delta^{2}}\sin^{2}\left(\sqrt{\kappa^{2}+\delta^{2}}z\right)\right]\\ I_{b}\left(z\right)=b\left(z\right)b^{*}\left(z\right) & = & I_{a}\left(0\right)\frac{\kappa^{2}}{\kappa^{2}+\delta^{2}}\sin^{2}\left(\sqrt{\kappa^{2}+\delta^{2}}z\right) \end{array}$$

where *κ* is the coupling coefficient and 2*δ* = *β_b_* − *β_a_* the difference between the phase constants of the two modes (*β* = 2*πn*/*λ*). The equations reveal the existence of a periodic exchange of signal between the two waveguides when they have the same propagation constant (*δ* = 0). The coupling period is twice the coupling length, *L_C_*, which is given by

(3) $$L_{C}=\frac{\pi }{2\sqrt{\kappa^{2}+\delta^{2}}}$$

**Figure 17:**
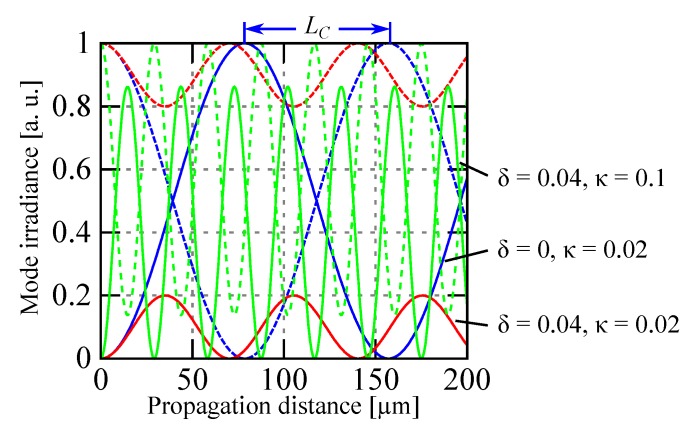
Coupling of energy between two propagation modes as a function of the coupling coefficient *κ* and the difference in propagation constant *δ*. In the three different cases (*δ* = 0 and *κ* = 0, blue lines; *δ* = 0.04 and *κ* = 0.02, red lines; *δ* = 0.04 and *κ* = 0.1, green lines), the dashed line represents the irradiance of mode a and the full line the irradiance of mode b with an irradiance at origin given by *I_a_*(0) = 1 and *I_b_*(0) = 0.

These relations are governed by the coupling coefficient, *κ*, which is proportional to the overlap between the two modes excited in the waveguides:

(4) $$\kappa \propto \;\;\int \;\;\;\;\;\int_{\;\;\;\;core}\;\;u\left(x,y\right)\;u\left(x+g+h,y\right)\;dx\;dy$$

where *u*(*x*, *y*) is the lateral profile of the mode, *g* the gap width, and *h* the thickness of the waveguide core. As we may see in Figure 17 if there is no phase constant difference (*δ* = 0) the coupling is ideal, and 100% of energy is exchanged after the length *L_C_*. However even a modest difference in phase constant (*δ* = 0.04 or about 1% change in effective index with *λ* ≈ 1500 nm and *n* ≈ 1.5) will spoil the coupling, which may be restored if we increase the coupling coefficient *κ*, here by a factor of 5, by bringing the two waveguides closer. We must note that the model is no more valid when the two modes are strongly coupled but the general behavior remains similar. The wavelength dependence of the evanescent coupler is one of its major drawback for broadband application even if some design may mitigate somewhat this issue.

The previous equations are essentially valid for both TE and TM polarization. However there is a difference in the coupling coefficient for the two polarizations that will translate in significant coupling length difference and in general we place a polarizer in front of the evanescent coupler. In order to obtain short coupling length, we have to achieve the largest possible ratio between the power flowing outside the core, which may excite the mode in the other waveguide, and the total transported power. Of course decreasing the gap will work but at constant gap width there are ways to keep the coupling distance short. The evanescent field decreases much slower in high index fluid than in air (lower index contrast) and a simple way to increase this ratio is to fill the gap between the two waveguides with a matching index fluid. Another way is to use specially designed waveguides, mostly presenting high refractive index contrast and sub-wavelength size, like the silicon nanowire waveguide [16], that intrinsically possess the ability to carry an important part of the power in the evanescent part of their fundamental propagation mode.

### 5.2. Principle Application

**Figure 18:**
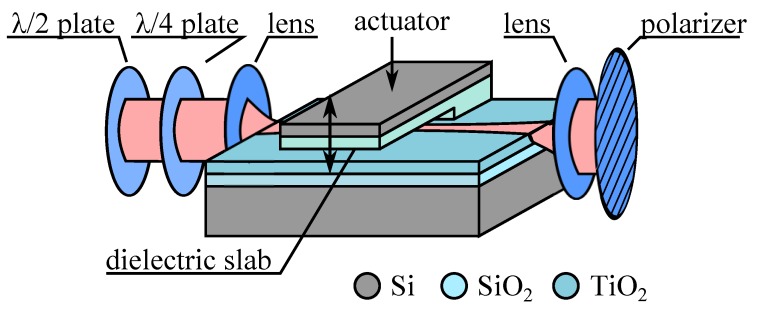
Schematic of operation of a displacement sensor using evanescent principle and a polarimetric interferometer [7] in a SiO_2_/TiO_2_ slab waveguide.

In the original proposal by Lukosz *et al.* based on effective index change, they made use of a very simple polarimetric interferometer, that is, they used the difference in effective index change for the two polarizations (Figure 18). The fabrication of the device was then very simple because they did not pattern a channel waveguide but instead used a slab waveguide. A rotating half-wave plate and a quarter-wave plate was used to transform the linearly polarized laser light for exciting the fundamental TE and TM mode with controlled amplitude and obtain the best fringe contrast. Then, the dielectric slab motion caused effective index changes for the two fundamental TE and TM modes, resulting in an overall phase shift between the two modes given by Δ*φ_TE/TM_* = 2*πL*/*λ*(Δ*N_TE_* − Δ*N_TM_*). Using a polarizer oriented at 45° at the waveguide output, the TE and the TM modes interfere and the resulting intensity change is directly linked to Δ*φ_TE/TM_* and thus to Δ*d*.

The authors called this use of the evanescent principle the “nanomechanical effect” as a Δ*d* of only 0.9 nm around an average gap of *d* = 35 nm was enough to induce a phase shift of *π* (a complete period of the interferometer response) for an interaction length of *L* = 1.2 mm at the wavelength of 632 nm. The actuator was build by hybrid integration of a Si/SiO_2_ structure, simply assembled by benefiting from reversible adhesive force appearing between the two dielectric layers. By applying voltage between the Si backbone of the actuator and the Si substrate below the waveguide the cantilevered slab could be brought in contact with the waveguide for modulating its effective refractive index. This basic idea was also used to build a microphone where the actuation was provided by the acoustical vibrations on a mylar sheet or a silicon membrane coupled to the dielectric slab [7,82,83,84].

The same group further developed this principle by building more complex circuits using rib channel waveguides. The 2D waveguide allowed more compact integration and they used Mach-Zehnder interferometer for building intensity modulator or combined them with multi-mode interference coupler for producing 2 × 2 optical switch [66].

Another team at Georgia Tech proposed the same type of devices relying on interaction with dielectric slab, but managed to build devices using stacked co-integration with polymer [8]. The movable polymer slab was built on a suspended polymer platforms electrostatically actuated and having protrusions below the surface for better contact with the waveguide. The process used multi-layer electroplated metal sacrificial layer.

Instead of using phase modulation, several groups proposed to use a slab coated with an absorptive metal [67,68,69,73]. Using this structure one team directly obtained an intensity modulator with about 35 dB contrast [68] with *L* = 2 mm long interaction. In both teams, the fabrication was based on hybrid assembly of two preprocessed silicon wafers, using SiON channel waveguide process. An older proposal by a team at TI made direct use of a metal membrane derived from the DMD projector chip for building this type of ON/OFF optical switch [54,72], but no demonstrator seem to have been built and the focus remained on the metal membrane development.

More recently, a similar device has been monolithically integrated on InP producing both phase and intensity modulators [41], with a projected use for co-integration with source and detector.

The evanescent principle has also been used for building optical switches, where the incoming signal is switched between two different outputs. The large number of devices operating on this principle follow one of two approaches: either a waveguide pair is fabricated in the coupled state and the actuator is used for spoiling the evanescent coupling or the waveguides are normally uncoupled and the actuator is used for inducing evanescent coupling.

**Figure 19:**
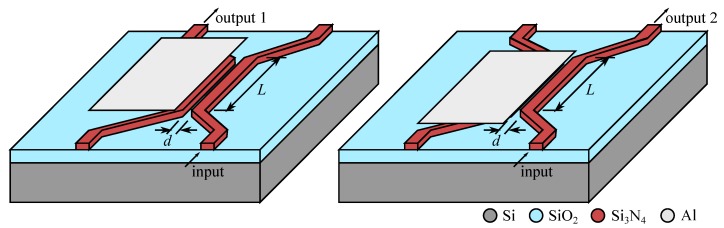
Schematic of operation of an optical switch based on spoiled evanescent coupling. (**Left**) In the initial state the light goes from input to output 1. (**Right**) The metal film spoils the coupling condition and the light goes straight and exits through output 2.

The original proposal at TI [54,72] used the first scheme and was supposed to use an absorptive metal membrane for spoiling the coupling condition, as shown in Figure 19. In this device the two coupled waveguides could be used for building 1 × 2 and 2 × 2 optical switches, however the tight tolerance for obtaining good coupler in passive material made this approach relatively challenging.

More recently, instead of a metallic film, a group used the motion of a dielectric in vicinity of one of the waveguide to change its effective index of refraction and again disrupt the coupling. Interestingly they used an in-plane configuration on a silicon platform, with a comb-drive actuator. At the difference of gap-closing actuators, the use of the comb-drive allowed for precise control of the coupling [23]. The device had a speed of 14 μs and presented a modest maximum extinction of 7 dB, presumably because the fabrication process (edge roughness) did not allow to decrease the gap below 150 nm.

A similar approach has been used with microring coupled waveguides, providing wavelength selective switch. The idea to use a ring resonator for obtaining a wavelength coupler or drop filter is as old as integrated optics [94] and in such device the microring is resonating at a particular wavelength that enables to couple the signal from the input waveguide to the ring and then to the output waveguide. The recent co-integration with an actuator adds the ability to tune this coupling in interesting manners.

**Figure 20:**
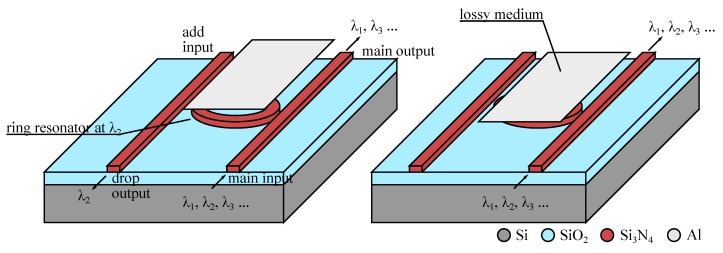
Schematic of operation of an add/drop wavelength switch based on waveguides coupled through a resonant ring [75]. (**Left**) The resonant ring channels the signal at wavelength *λ*_2_ to the drop output. (**Right**) A lossy medium perturbs the evanescent field spoiling the ring resonance and *λ*_2_ goes to the main output.

The simplest technique consists in spoiling the coupling by making the resonant ring lossy. As can be seen in Figure 20, it is sufficient to bring into the evanescent field of the ring a broadband light absorbing material (here a metal layer in aluminum) to lower the quality factor of the ring resonator and avoid any coupling out of the input waveguide. In this way we get a switchable drop filter, able to get at will one channel from a multichannel signal, depending on the ring resonant wavelength (changing with radius and index of refraction). We note that the channel at *λ*_2_ may even be coupled at the add input and it will be re-injected in the multichannel signal to the main output. The fabrication process is based on stacked integration with high index ridge waveguides as shown in Figure 5 and requires nano-lithography for precisely defining the ring close to the waveguides [74,75].

In a second technique, the coupling is induced by the actuator, by narrowing the gap between the normally uncoupled elements, effectively increasing the coupling coefficient *κ*. This configuration has the advantage that if a continuous control of the gap is available, a precise control of *κ* will allow placing the operation point anywhere from 0% to 100% transfer of energy.

**Figure 21:**
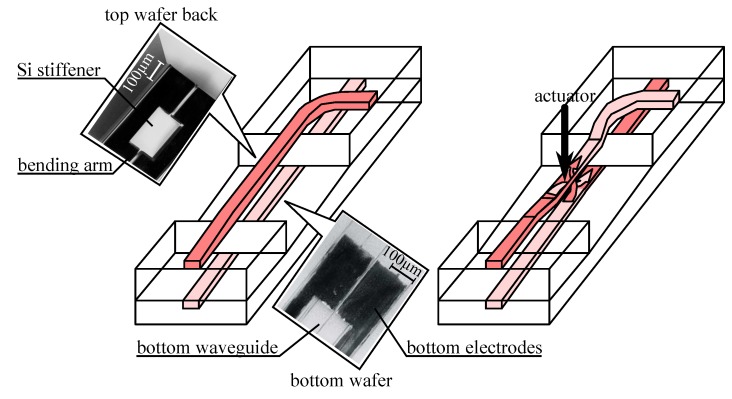
Principle of operation of 2 × 2 optical switch using evanescent coupling between a bending and a fixed waveguide [73] (insets: view of the back of the top waveguide supporting structure and view of the bottom electrode and waveguide).

Early devices exploiting this principle were built by hybrid assembly of two silicon chips using Si_3_N_4_ waveguides [73]. The high contrast waveguide gave a significant evanescent field for obtaining short coupling, and helped reduce the device size. As shown in Figure 21, one of the two waveguides is fixed on the lower chip and the other waveguide on the top chip is placed on a suspended bridge with a rigid central platform that would allow better contact between the two waveguides. Planar electrodes on both side of the two waveguide form a gap-closing actuator that is used for bringing the waveguides into contact, unfortunately without providing a proportional actuation. The measurements done with the device were not conclusive, presumably because of this particular drawback of the vertical actuation scheme.

A similar optical switch was proposed on InP/InGaAsP [39] but using an in-plane configuration, using electrostatic actuation to attract the two waveguides in a gap closing configuration. Because of the small gap (2 μm), the speed of such MEMS switch was rather high and reached 10 μs, and was even faster during voltage release. It was noted that the evanescent field did not extend as far as the original 2 μm gap and an optimized geometry with smaller gap should reach a speed of a few μs. Other development also occurred on high-index contrast silicon platform [22]. These devices tried to circumvent the issue linked with the gap closing actuator and used in-plane actuator based on comb-drive, which allows proportional actuation. Moreover, in this case it is possible to fabricate the waveguides further apart with a larger gap *d* to relax the fabrication constraint and then, by biasing the actuator at a fixed voltage, to narrow the gap until the coupling just disappears, insuring optimal actuator course and speed.

To avoid the snapping effect linked with the gap closing actuator, a group at the University of Southampton has recently proposed to use electrostatic repulsive force instead of attractive force. By polarizing the two waveguide at the same potential (with respect to a ground plane), the waveguide will repel each other continuously with increased voltage [37]. This behavior force to define a narrow gap between the two waveguides at fabrication, where the coupling will be maximum. The authors simulations show that the device could also be used as an efficient optical buffer delay by tuning phase and group velocity [98].

**Figure 22:**
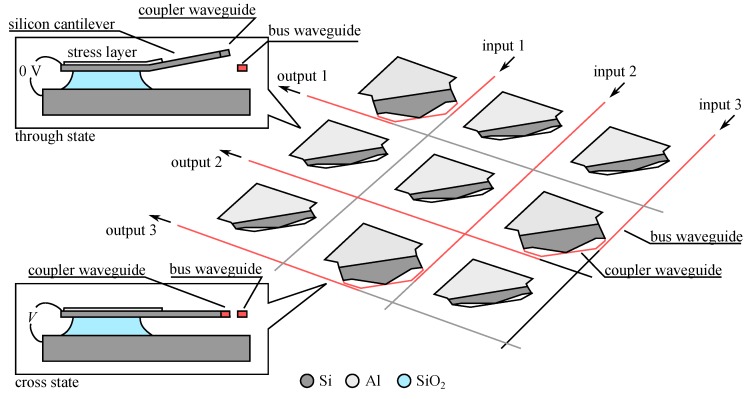
Principle of operation of 3 × 3 non-blocking optical switch matrix using movable evanescent coupler on a bending actuator (adapted from [29]) (inset top: cross-section of the switch cell in the through state showing the bending due to the stressed metal layer) (inset bottom: cross-section of the switch cell in the cross state after applying a voltage *V*).

The integration capabilities offered by the SOI platform with sub-wavelength waveguides allow ambitious realization as proposed in different groups across the world. Probably the largest structure based on this technology so far is the optical switch matrix proposed by UCLA [29]. The device is based on an optical switch with evanescent coupling, but they proposed an original actuation scheme based on a bending actuator. As we see in Figure 22 the matrix is composed of identical elements allowing coupling between series of input and output waveguides. The team built matrix with 50 inputs and 50 outputs that measured less than 8 mm × 8 mm, smaller than the best open-space switch matrix produced so-far [13]. In the through state, the cell is powered-off and the coupler waveguide is kept far from the bus waveguide because of the bend in the silicon cantilever supporting the coupler waveguide. The bend is induced by the gradient of stress between the sputtered metal film and the silicon thin-film of the SOI wafer. As the electrostatic actuator is powered, the coupler waveguide is attracted toward the substrate and reach best coupling position, allowing the signal to be coupled from the input bus waveguide to the coupler waveguide on one side and from the coupler waveguide to the output bus waveguide on the other side. The loss of one unit in the through state is about 0.04 dB, and it is much larger in the cross-state with 2.47 dB. However, as can be seen in the figure, whatever the number of inputs and outputs there is only one switch in the cross-state, which is an advantage of this matrix architecture over matrix built from cascaded 2 × 2 optical switches [73]. The switching voltage was 14 V with a speed around 3.8 μs and an extinction ratio of about 26 dB between the through and cross states. The useful bandwidth of the evanescent coupler was estimated at 13 nm, limiting the use of this device in broadband application.

**Figure 23:**
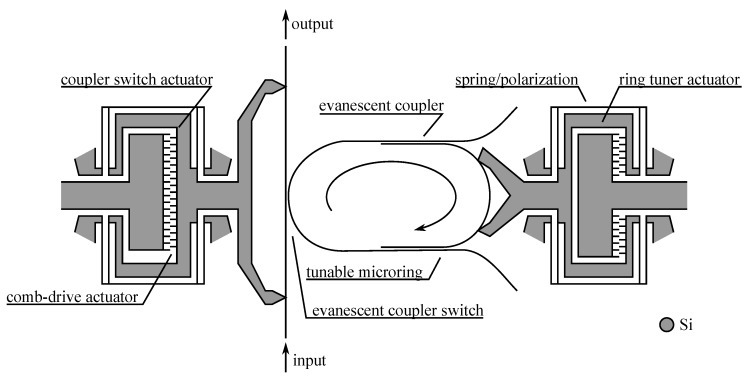
Schematic of a tunable microring resonator with ON/OFF coupling capability (adapted from [25]).

The prolific group at Tohoku University has also proposed very interesting devices where they are starting to integrate multiple optical functions together on the same device. The possibility to provide tunable wavelength filter is important for multiple integrated optics applications, and the group proposed a simple approach based on the continuous control of the optical path length in a microring resonator while providing at the same time a possibility to turn off this feature altogether [25]. The basic structure is relatively simple but nicely demonstrates the technological mastery attained on this platform. In Figure 23, the system works between input and output as a simple drop wavelength filter, suppressing the wavelength at the microring resonance with a quality factor of about 4500. The right comb-drive actuator may continuously change the length of the ring, directly modifying the resonant wavelength of the microring. In this device a motion of 1 μm of the actuator resulted in a shift of 10 nm around the wavelength of 1500 nm, providing an interesting tunable drop filter. The team thought that during the tuning of the resonator the system would continuously scan all the intermediate wavelengths, and they added a feature to fully uncouple the microring from the main waveguide. In fact, the left actuator may move the main waveguide by more than 1 μm, effectively uncoupling the microring. By using this actuator first, it becomes simple to jump from one wavelength to another without scanning through the intermediate wavelength. The device was built using the high index contrast Si platform with nanowire waveguide resulting in a very compact system with dimensions of only 150 μm × 80 μm.

The same structure for tuning optical path length, but without the closed ring, has also been used for a simple delay tuner, providing a maximum phase shift of 2*π* with 31 V [24].

**Figure 24:**
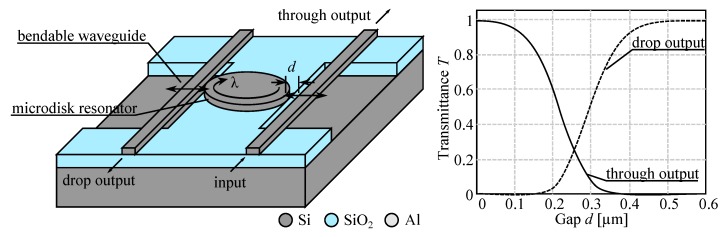
(**Left**) Schematic of operation of a silicon microdisk resonator wavelength switch. (**Right**) Theoretical response of the drop filter as a function of the gap at *λ*, the resonant wavelength of the microdisk.

Coupling to a microdisk using variation of the gap between the waveguides for tuning has also been used for wavelength selective switches [16]. In the early version shown in Figure 24, the actuation is in-plane and the process requires only one active layer and few lithography steps. This simplicity of operation unfortunately make strong coupling harder to achieve, giving a modest quality factor of 7700 and an extinction coefficient of 9 dB at the resonant wavelength *λ*. The same team rapidly proposed an out-of-plane actuation scheme that gave much better results. In that case the microdisk and the waveguides are not in the same plane and the coupling happens between the top and bottom surfaces, increasing the coupling area and decreasing the surface roughness. The electrostatic actuator used recessed electrodes for providing proportional actuation in a relatively long range, and for almost continuously tuning the device. In addition to offer wavelength selective switch behavior, the device presented tunable dispersion and delay [17]. Actually, by varying the gap spacing between the waveguide and the disk this microresonator can dynamically operate in either under-, critical or over-coupling regime resulting in variation of group delay from 27 ps to 65 ps, and group velocity dispersion from 185 ps/nm to 1200 ps/nm. The waveguide transmittance is suppressed by 30 dB in critical coupling, and the quality factor of the microdisk is measured to be as high as 10^5^.

This type of device presenting wavelength selectivity relies on the quality factor of the resonator [99] and besides microdisks, Si microrings with sub μm waveguide width [19] and microtoroidal resonators have also been tried as they may possess even lower loss [20,21]. The results demonstrated so far tend to give the advantage to the microdisk architecture, but more work is occurring on this architecture that may modify this evaluation in the future.

The sensitivity of the resonator is not only useful for optical telecommunication but also for high resolution sensing. In an interesting evolution of the microring resonator a team at Yale has used the technique to measure the nano-displacement due to the optical force in the ring itself. The team used a slot waveguide, that is a waveguide composed of two sub-wavelength high-index beams with a sub-wavelength gap in between. In practice the waveguides were etched in a 220 nm thick silicon layer, with two beams of width 350 nm and an air gap of 80 nm. This waveguide caries most of the optical power in the air gap between the two beams, and creates a strong optical gradient that creates optical forces pulling the beams toward each others. The beams are released along a 2 μm short section of the ring where they are able to vibrate and modify the resonant wavelength of the ring. The ring had a quality factor of more than 60,000 allowing to reach a displacement sensitivity of 0.45 fm/$\sqrt{Hz}$.

**Figure 25:**
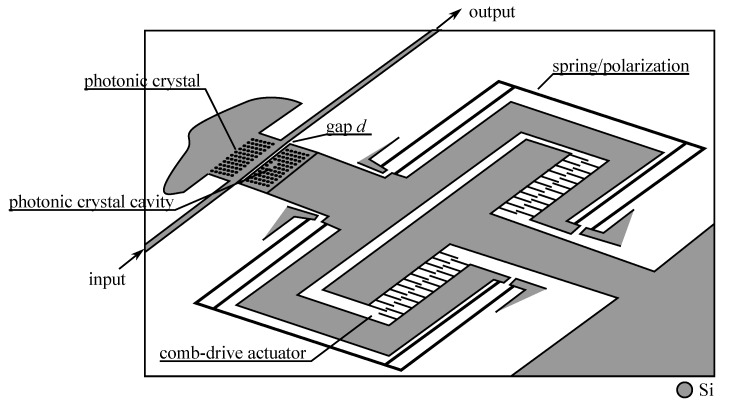
Schematic of a drop wavelength filter coupling a photonic crystal resonant cavity with a photonic waveguide.

For ultimate compactness, instead of using the previous micro-ring/disk/toroidal resonators, one may use photonic crystal. A team at Tohoku University proposed to build a wavelength drop filter (notch filter) by coupling a waveguide to a resonant cavity composed of a few missing elements in the photonic crystal lattice (Figure 25). The complete device is built on the device layer of a SOI wafer with an integrated comb-drive actuator [32]. When the gap *d* is small enough and coupling between the waveguide and the cavity occurs, the cavity emits light perpendicular to the device plane, decreasing the transmitted intensity accordingly. They showed that best performance was obtained when the gap was below 200 nm, resulting in a drop efficiency of 18 dB at a wavelength of 1.5724 μm, the resonant wavelength of the cavity. The filter presented a bandwidth of 0.9 nm, close to the designed value of 0.75 nm.

## 6. Devices Based on Change of the Waveguide Strain

### 6.1. Design Consideration

Waveguide strain created by an actuator (for an active device, internally powered or, for a sensor, powered by the energy of the measurand) will have two effects: inducing stress in the waveguide and changing its length. The induced stress in the waveguide is converted to refractive index change through the elasto-optic effect effectively changing the phase of the propagating mode [4]. Similarly, the change in propagation distance (length increase) will also change the light phase [79]. The relative influence of both effects may generally be controlled by using specific materials and by precisely positioning the waveguide on bending mechanical element. For example, if we consider a waveguide passing on membrane we find different cases, depending on the position of the waveguide in the middle or on the side of the membrane.

It has been shown that the deflection of a membrane with length *a*, width *b* and thickness *t* ≪ *a*, *b* is maximized when *a*/*b* = 2. By placing the waveguide in the center of such membrane and for a deflection of *h* ≪ *a*, the change in optical phase is maximal and is given by [79]:

$$\Delta \phi =\frac{2\pi }{\lambda }\frac{N\pi^{2}}{4}\frac{h^{2}}{a}$$

In the other hand the elasto-optic effect is maximized at the position of maximum stress, that is on the side of the membrane, and is given more generally by [4]:

$$\Delta \phi =\frac{2\pi \nu \epsilon_{0}}{4}\int_{0}^{a}\int \int E_{i}^{*}\Delta n_{i}E_{i}\;dx\;dy\;dz$$

where *E_i_* is the relevant optical field for the TE or TM modes, and Δ*n_i_* the change in refractive index due to the stress in the material (we have $\Delta n_{i}=-\frac{1}{2}n^{3}p_{ij}S_{j}$ using *p_ij_* the elasto-optic tensor and S_*j*_ the stress vector component). It is interesting to note that the elasto-optic index change may be positive or negative depending on the position of the waveguide on the membrane. Precisely designing this type of devices is somewhat tricky if the elasto-optic effect is dominating the behavior because, if the photoelastic coefficients of many bulk optical materials are well known, those of thin films are not as their structural and compositional properties depend on the deposition methods [78].

By using very small deflection it is relatively simple to obtain devices with dominant elasto-optic effect [4], but proper use of material and geometry has also been used for building devices where the geometrical effect was dominant [79]. Finally, in practical devices, the induced phase change is generally converted to intensity modulation using some sort of two-waves interferometric arrangement.

### 6.2. Principle Application

**Figure 26:**
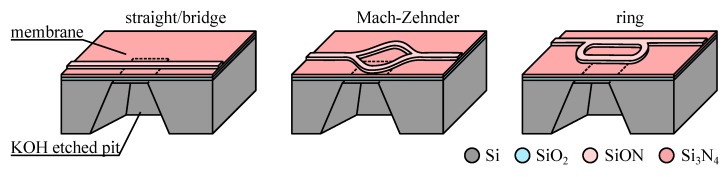
Schematic of strain based optical micromachined pressure sensors using strip loaded lateral confinement with (**left**) a straight waveguide [51], (**center**) a Mach-Zehnder interferometer [4] and (**right**) a ring resonator [70].

The first application of this principle used a Mach-Zehnder arrangement to convert the phase change in intensity variation. The device, shown in Figure 26 placed the SiO_2_/glass waveguide on the side of a diaphragm etched from the backside with EDP and an electrochemical etch-stop. The diaphragm thickness was about 7 μm, with 5 μm of silicon EPI layer and the rest for the SiO_2_ and glass layers for the waveguide. The second arm of the interferometer was placed on the unetched silicon substrate and is used as a reference signal [4]. The half-wave pressure, where the phase shift is *π*, was obtained with a pressure of 0.8 atm (80 kPa) resulting in an extinction of 10 dB.

Another team improved on this original device by first integrating a photodiode in the silicon substrate and, by working at the He-Ne laser wavelength of 0.632 μm, to directly measure the light at the Mach-Zehnder output [77]. Then, taking benefit of the change of opposite signs in refractive index if the waveguides are properly placed on the membrane, they improved the pressure sensor sensitivity [78]. In practice, by placing one arm on the membrane center and the second arm on its side, they obtained half-wave pressure as low as 2.7 kPa, with, it is true, a smaller membrane thickness *t* = 5 μm. Moreover, they confirmed the higher sensitivity obtained for the TM modes, and showed that layers under compressive stress have better performance (particularly under longitudinal stress), giving an advantage to the PECVD layers over the LPCVD layers for building the waveguide. A team at University of Waterloo used ARROW waveguide instead of the rib waveguides generally used by others, and observed similar behaviors, including a doubling in sensitivity when one waveguide was placed on the edge and one at the center in a push-pull configuration [71].

Another similar pressure sensor has been proposed but based on an original interferometric scheme using an imbalanced Mach-Zehnder interferometer. In their design the measurand (here the pressure) is not encoded in the intensity at the output of the interferometer, but in the delay between coherent wave packet obtained with a broadband source with a coherence length of only 30 μm. A fixed delay is imposed at design by using a Mach-Zehnder with arm length difference longer than this value, preventing the emergence of interference at the interferometer output. The pressure is slightly changing the delay value, which can then be retrieved by using either an optical spectrum analyzer or another interferometer with the same fixed delay between its two arms as demodulator [79]. Interestingly in their device the photoelastic effect could be almost completely neglected and only the geometric effect appears. This may probably be traced to their use of layers in tensile stress for building the waveguides, reducing the effect of photoelasticity [77].

As with evanescent interaction with a dielectric slab, the simplest approach for using effective refractive index change consists in using the difference existing between the TE and the TM modes in a single waveguide and use a polarimetric interferometer. This scheme (Figure 26-left) has been used recently for exploring scaling rules for pressure sensors [53]. The same group that pioneered the technology, showed that by maintaining the ratios *a*/*b* and *a*^3^/*t*^2^, the pressure sensitivity could be kept constant. They also demonstrated the use of such structure as acceleration sensor by adding a proof mass in the center of the membrane back [52]. They experimentally studied the effect of the membrane thickness and of the waveguide position on the acceleration sensitivity, obtaining about 93 mrad/g for a membrane size 10 mm × 10 mm × 50 μm with a central proof-mass 5 mm × 5 mm × 300 μm micromachined in silicon with PolyStyrene/SiO_2_ inverted rib waveguides atop the membrane. An earlier work [51] on straight waveguide placed atop micromachined bridge may be based on the same principle but the author are not clear enough and appears to base the observed effect on bending induced loss.

Other type of phase sensing mechanism can also be employed for taking benefit of this principle. For example, instead of looking at intensity modulation with a Mach-Zehnder or a polarimetric interferometer, we have already seen that delay modulation can be used, but also frequency modulation. In fact, the frequency shift of a resonator is considered as one of the best method for measurement, as frequency may be easily measured with precision well below 10^9^. Early work on this principle was performed by coupling a ring resonator to a straight waveguide (Figure 26-right) and detecting the shift in resonant wavelength when the effective refractive index is changed by the pressure on the diaphragm under the ring [70]. The detection is obtained by sweeping the laser diode current that result in a regular sweep in wavelength. After calibration the current shift between the start and the point of minimum transmissivity may be directly linked to a particular phase shift in the ring. This interrogation method provides a high degree of immunity to changes in coupling losses in the optical circuit (connector, lot-to-lot optical absorption variation, *etc.*). They obtained again a much higher sensitivity with TM mode than with TE mode, giving a half-wave pressure of about 320 kPa for TM mode, and 23 times higher with the TE mode. The device was unfortunately found to be highly sensitive to temperature with a half-wave temperature of less than 1 °C preventing high precision measurement. Still, the temperature was affecting TE and TM modes equivalently opening the possibility to discriminate between the two effects without requiring complex temperature stabilization.

## 7. Conclusions

From the beginning, integrated optics and MEMS technology were poised to converge, but it took years before this chance started to materialize at the dawn of the 1990s. Since then, many groups have explored multiple technologies, multiple scheme of co-integration and multiple applications from optical telecommunications to ultimate sensing.

Still if the available literature on the field of optical MEMS is large and if multiple products (display array, micro-spectrometer, optical scanners, optical fiber switches...) have already emerged on the market, it is much less true for devices based on waveguide and MEMS actuators co-integration. The complexity of bringing such components to market is real, principally because of the complex fabrication and packaging while the cost issue is exacerbated by the large chip dimensions imposed by most waveguide technologies.

However the high refractive index contrast silicon platform [85] based on nanowire waveguide possess the qualities to be a solid candidate for new and commercial development based on waveguide and actuator co-integration: the high contrast provides compact devices, the coupling loss with optical fiber using taper is reasonably low, the evanescent field is strong, the silicon layer is easily transformed in an efficient actuator and it has demonstrated very promising integration capabilities. For industry, it is not clear that there is room for other technologies, because, as microelectronics has proved, the emergence of a “standard” compact technology is key in pushing devices out of the labs.

For the longer perspective and for researchers the opportunities do not stop here, and new directions are still emerging for example toward co-integration of actuators with plasmonic waveguide [50,100], resulting in switch and modulators with extreme compactness of the order 0.5 μm × 2 μm !

To paraphrase a famous paper [101], we could say, that in waveguide and MEMS actuator co-integration there is still plenty of room at the bottom !

## References

[1] Miller S. (1969). Integrated optics: An introduction. Bell Syst. Tech. J..

[2] Nathanson H., Wickstrom R. (1965). A resonant-gate silicon surface transistor with high-Q bandpass properties. Appl. Phys. Lett..

[3] Maluf N. (1999). Foreword. An Introduction to Microelectromechanical Systems Engineering.

[4] Ohkawa M., Izutsu M., Sueta T. (1989). Integrated optic pressure sensor on silicon substrate. Appl. Opt..

[5] Hogari K., Matsumoto T. (1991). Electrostatically driven micromechanical 2X2 optical switch. Appl. Opt..

[6] Bezzaoui H., Voges E. (1991). Integrated optics combined with micromechanics on silicon. Sens. Actuators A Phys..

[7] Lukosz W., Pliska P. (1991). Integrated optical interferometer as a light modulator and microphone. Sens. Actuators A Phys..

[8] Kim Y., Allen M., Hartman N. (1992). Micromechanically based integrated optic modulators and switches. Proc. SPIE.

[9] Marxer C., Thio C., Grétillat M.A., de Rooij N., Bättig R., Anthamatten O., Valk B., Vogel P. (1997). Vertical Mirrors Fabricated by Deep Reactive Ion Etching for Fiber-Optic Switching Applications. J. Microelectromech. Syst..

[10] Toshiyoshi H., Fujita H. (1996). Electrostatic Micro Torsion Mirrors for an Optical Switch Matrix. J. Microelectromech. Syst..

[11] Roussey M., Bernal M.P., Courjal N., Van Labeke D., Baida F.I., Salut R. (2006). Electro-optic effect exaltation on lithium niobate photonic crystals due to slow photons. Appl. Phys. Lett..

[12] Tabib-Azar M., Beheim G. (1997). Modern trends in microstructures and integrated optics for communication, sensing, and actuation. Opt. Eng..

[13] Solgaard O., Godil A.A., Howe R.T., Lee L.P., Peter Y.A., Zappe H. (2014). Optical MEMS: From Micromirrors to Complex Systems. J. Microelectromech. Syst..

[14] Marcuse D. (1972). Light Transmission Optics.

[15] Duguay M.A., Kokubun Y., Koch T.L., Pfeiffer L. (1986). Antiresonant reflecting optical waveguides in SiO_2_-Si multilayer structures. Appl. Phys. Lett..

[16] Lee M.C., Wu M. (2005). MEMS-actuated microdisk resonators with variable power coupling ratios. IEEE Photon. Technol. Lett..

[17] Lee M.C.M., Wu M.C. (2006). Tunable coupling regimes of silicon microdisk resonators using MEMS actuators. Opt. Express.

[18] Nawrocka M.S., Liu T., Wang X., Panepucci R.R. (2006). Tunable silicon microring resonator with wide free spectral range. Appl. Phys. Lett..

[19] Takahashi K., Kanamori Y., Kokubun Y., Hane K. (2008). A wavelength-selective add-drop switch using silicon microring resonator with a submicron-comb electrostatic actuator. Opt. Express.

[20] Yao J., Leuenberger D., Lee M.C.M., Wu M. (2007). Silicon Microtoroidal Resonators With Integrated MEMS Tunable Coupler. IEEE J. Sel. Top. Quantum Electron..

[21] Yao J., Wu M.C. (2009). Bandwidth-tunable add-drop filters based on micro-electro-mechanical-system actuated silicon microtoroidal resonators. Opt. Lett..

[22] Akihama Y., Kanamori Y., Hane K. (2011). Ultra-small silicon waveguide coupler switch using gap-variable mechanism. Opt. Express.

[23] Chatterjee R., Wong C.W. (2010). Nanomechanical Proximity Perturbation for Switching in Silicon-Based Directional Couplers for High-Density Photonic Integrated Circuits. J. Microelectromech. Syst..

[24] Ikeda T., Takahashi K., Kanamori Y., Hane K. (2010). Phase-shifter using submicron silicon waveguide couplers with ultra-small electro-mechanical actuator. Opt. Express.

[25] Ikeda T., Kanamori Y., Hane K. Si photonic nano-wire tunble micro-ring resonator composed of triply-liked variable couplers. Proceedings of 2012 IEEE 25th International Conference on Micro Electro Mechanical Systems (MEMS 2012).

[26] Baumann F., Chan H., Fuchs D., Stuart H. Monolithic waveguide/MEMS switch. U.S. Patent.

[27] Fuchs D., Chan H., Stuart H., Baumann F., Greywall D., Simon M., Wong-Foy A. (2004). Monolithic integration of MEMS-based phase shifters and optical waveguides in silicon-on-insulator. Electron. Lett..

[28] Fuchs D., Doerr C., Aksyuk V., Simon M., Stulz L., Chandrasekhar S., Buhl L., Cappuzzo M., Gomez L., Wong-Foy A. (2004). A Hybrid MEMS-Waveguide Wavelength Selective Cross Connect. IEEE Photon. Technol. Lett..

[29] Han S., Seok T.J., Quack N., Yoo B.W., Wu M.C. (2015). Large-scale silicon photonic switches with movable directional couplers. Optica.

[30] Zandi K., Belanger J.A., Peter Y.A. (2012). Design and Demonstration of an In-Plane Silicon-on-Insulator Optical MEMS Fabry-Pérot-Based Accelerometer Integrated With Channel Waveguides. J. Microelectromech. Syst..

[31] Bulgan E., Kanamori Y., Hane K. (2008). Submicron silicon waveguide optical switch driven by microelectromechanical actuator. Appl. Phys. Lett..

[32] Kanamori Y., Takahashi K., Hane K. (2009). An ultrasmall wavelength-selective channel drop switch using a nanomechanical photonic crystal nanocavity. Appl. Phys. Lett..

[33] Li M., Pernice W.H.P., Tang H.X. (2010). Ultrahigh-frequency nano-optomechanical resonators in slot waveguide ring cavities. Appl. Phys. Lett..

[34] Bakke T., Tigges C., Lean J., Sullivan T., Spahn O. (2002). Planar microoptomechanical waveguide switches. IEEE J. Sel. Top. Quantum Electron..

[35] Pruessner M., Kelly D., Datta M., Lim H., Maboudian R., Ghodssi R. Design and fabrication of an InP-based moving waveguide 1 × 2 optical MEMS switch. Proceedings of 2003 International Semiconductor Device Research Symposium.

[36] Pruessner M.W., Siwak N., Amarnath K., Kanakaraju S., Chuang W.H., Ghodssi R. (2006). End-coupled optical waveguide MEMS devices in the indium phosphide material system. J. Micromech. Microeng..

[37] Podoliak N., Ng W.H., Liu H., Kenyon A.J., Stewart W., Horak P. MEMS actuation for a continuously tunable optical buffer. Proceedings of 2014 International Conference on Optical MEMS and Nanophotonics.

[38] Kelly D., Pruessner M., Amarnath K., Datta M., Kanakaraju S., Calhoun L., Ghodssi R. (2004). Monolithic Suspended Optical Waveguides for InP MEMS. IEEE Photon. Technol. Lett..

[39] Pruessner M., Amarnath K., Datta M., Kelly D., Kanakaraju S., Ho P.T., Ghodssi R. (2005). InP-based optical waveguide MEMS switches with evanescent coupling mechanism. J. Microelectromech. Syst..

[40] Pruessner M.W., Stievater T.H., Khurgin J.B., Rabinovich W.S. (2011). Integrated waveguide-DBR microcavity opto-mechanical system. Opt. Express.

[41] Pruessner M., Park D., Stievater T., Kozak D., Rabinovich W. (2014). An Optomechanical Transducer Platform for Evanescent Field Displacement Sensing. IEEE Sens. J..

[42] Siwak N.P., Fan X.Z., Ghodssi R. (2015). Fabrication challenges for indium phosphide microsystems. J. Micromech. Microeng..

[43] Guerre R., Fahrni F., Renaud P. (2006). Fast 10-μs Microelectromechanical Optical Switch Inside a Planar Hollow Waveguide (PHW). J. Lightwave Technol..

[44] Brown K.S., Taylor B.J., Dawson J.M., Hornak L.A. (1998). Polymer waveguide cointegration with microelectromechanical systems (MEMS) for integrated optical metrology. Proc. SPIE.

[45] Dickey F.M., Holswade S.C., Hornak L.A., Brown K.S. (1999). Optical methods for micromachine monitoring and feedback. Sens. Actuators A Phys..

[46] Hornak L., Famouri P., Dawson J., Wang L., Ghaffarian R. (2001). MOEMS Integrated Optical Monitoring. Proc. SPIE.

[47] Dawson J., Wang L., McCormick W., Rittenhouse S., Famouri P., Hornak L. (2003). Integrated Optical Monitoring of MEMS for Closed-Loop Control. Proc. SPIE.

[48] Bakke T., Tigges C., Sullivan C. (2002). 1 × 2 MOEMS switch based on silicon-on-insulator and polymeric waveguides. Electron. Lett..

[49] Liu H.B., Chollet F. (2009). Moving Polymer Waveguides and Latching Actuator for 2 × 2 MEMS Optical Switch. J. Microelectromech. Syst..

[50] Dennis B.S., Haftel M.I., Czaplewski D.A., Lopez D., Blumberg G., Aksyuk V.A. (2015). Compact nanomechanical plasmonic phase modulators. Nat. Photon..

[51] Wu S., Frankena H.J. (1993). Integrated optical sensors using micromechanical bridges and cantilevers. Proc. SPIE.

[52] Saito N., Miura Y., Oshima T., Ohkawa M., Sato T. (2013). Experimental study of sensitivity dependences on waveguide position and diaphragm thickness in silicon-based guided-wave optical accelerometer. Opt. Eng..

[53] Ohkawa M., Sato T. (2012). Scale-reduction rule without drop in the sensitivity of a silicon-based guided-wave optical pressure sensor using a micromachined diaphragm. Opt. Eng..

[54] Boysel R.M., McDonald T.G., Magel G.A., Smith G.C., Leonard J.L., Tabib-Azar M., Polla D.L. (1993). Integration of deformable mirror devices with optical fibers and waveguides. Proc. SPIE.

[55] Kobayashi D., Okano H., Horie M., Otsuki H., Sato K., Horino M. PLC-based micromechanical optical switch with magnetic drive. Proceedings of 1997 IEEE/LEOS International Conference on Optical MEMS and Their Applications.

[56] Kobayashi D., Okano H., Otsuki H., Sato K., Horino M. Magnetically driven micromechanical optical switch. Proceedings of 1997 Pacific Rim Conference on Lasers and Electro-Optics (CLEOPR-97).

[57] Ollier E., Labeye P., Revol F. (1995). Micro-opto mechanical switch integrated on silicon. Electron. Lett..

[58] Ollier E., Mottier P. (1996). Integrated electrostatic micro-switch for optical fibre networks driven by low voltage. Electron. Lett..

[59] Ollier E., Labeye P., Mottier P. (1997). Integrated micro-opto-mechanical vibration sensor connected to optical fibres. Electron. Lett..

[60] Ollier E., Philippe P., Chabrol C., Mottier P. (1999). Micro-opto-mechanical vibration sensor integrated on silicon. J. Lightwave Technol..

[61] Ollier E., Chabrol C., Enot T., Brunet-Manquat P., Margail J., Mottier P. 1 × 8 micro-mechanical switches based on moving waveguides for optical fiber network switching. Proceedings of 2000 IEEE/LEOS International Conference on Optical MEMS.

[62] Ollier E. (2002). Optical MEMS devices based on moving waveguides. IEEE J. Sel. Top. Quantum Electron..

[63] Makihara M., Sato N., Shimokawa F., Nishida Y. (1999). Micromechanical optical switches based on thermocapillary integrated in waveguide substrate. J. Lightwave Technol..

[64] Fouquet J. Compact optical cross-connect switch based on total internal reflection in a fluid-containing planar lightwave circuit. Proceedings of 2000 Optical Fiber Communication Conference.

[65] Ollier E., Divoux C., Margail J., Enot T., Ortiz L., Gobil Y., Salhi M., Berruyer P., Gliere A., Bontemps A., Laporte M., Bruel M. Electrostatically actuated micro-fluidic optical cross-connect switch. Proceedings of 2003 IEEE/LEOS International Conference on Optical MEMS.

[66] Dangel R., Lukosz W. (1998). Electro-nanomechanically actuated integrated-optical interferometric intensity modulators and 2 × 2 space switches. Opt. Commun..

[67] Gui C., Veldhuis G.J., Koster T.M., Lambeck P.V., Berenschot J.W., Gardeniers J.G.E., Elwenspoek M. (1999). Fabrication of nanomechanical optical devices with aligned wafer bonding. Microsyst. Technol..

[68] Veldhuis G.J., Gui C., Nauta T., Koster T.M., Berenschot J.W., Lambeck P.V., Gardeniers J.G.E., Elwenspoek M. (1998). Mechano-optical waveguide on-off intensity modulator. Opt. Lett..

[69] Veldhuis G., Nauta T., Gui C., Berenschot J., Lambeck P. (1999). Electrostatically actuated mechanooptical waveguide ON-OFF switch showing high extinction at a low actuation-voltage. IEEE J. Sel. Top. Quantum Electron..

[70] de Brabander G., Boyd J., Beheim G. (1994). Integrated optical ring resonator with micromechanical diaphragms for pressure sensing. IEEE Photon. Technol. Lett..

[71] Benaissa K., Nathan A. (1995). ARROW-based integrated optical pressure sensors. Proc. SPIE.

[72] Magel G. (1996). Integrated optic devices using micromachined metal membrane. Proc. SPIE.

[73] Chollet F., de Labachelerie M., Fujita H. (1999). Compact evanescent optical switch and attenuator with electromechanical actuation. IEEE J. Sel. Top. Quant. Electron..

[74] Nielson G.N., Seneviratne D., Lopez-Royo F., Rakich P.T., Giacometti F., Tuller H.L., Barbastathis G. MEMS based wavelength selective optical switching for integrated photonic circuits. Proceedings of 2004 Conference on Lasers and Electro-Optics.

[75] Nielson G., Seneviratne D., Lopez-Royo F., Rakich P., Avrahami Y., Watts M., Haus H., Tuller H., Barbastathis G. (2005). Integrated wavelength-selective optical MEMS switching using ring resonator filters. IEEE Photon. Technol. Lett..

[76] Brière J., Beaulieu P.O., Saidani M., Nabki F., Menard M. (2015). Rotational MEMS mirror with latching arm for silicon photonics. Proc. SPIE.

[77] Fischer K., Müller J. (1992). Sensor application of SiON integrated optical waveguides on silicon. Sens. Actuators B Chem..

[78] Fischer K., Muller J., Hoffmann R., Wasse F., Salle D. (1994). Elastooptical properties of SiON layers in an integrated optical interferometer used as a pressure sensor. J. Lightwave Technol..

[79] Porte H., Gorel V., Kiryenko S., Goedgebuer J.P., Daniau W., Blind P. (1999). Imbalanced Mach-Zehnder Interferometer Integrated in Micromachined Silicon Substrate for Pressure Sensor. J. Lightwave Technol..

[80] Gorecki C., Chollet F., Kawakatsu H., Fujita H. (1997). Silicon-based integrated interferometer with phase modulation driven by surface acoustic waves. Opt. Lett..

[81] Hoffmann M., Bezzaoui H., Voges E. (1994). Micromechanical cantilever resonators with integrated optical interrogation. Sens. Actuators A Phys..

[82] Lukosz W., Pliska P. (1992). Integrated optical nanomechanical light modulators and microphones. Sens. Mater..

[83] Pliska P., Lukosz W. (1993). Electrostatically actuated integrated optical nanomechanical devices. Proc. SPIE.

[84] Pliska P., Lukosz W. (1994). Integrated-optical acoustical sensors. Sens. Actuators A Phys..

[85] Yamada H., Chu T., Ishida S., Arakawa Y. (2006). Si Photonic Wire Waveguide Devices. IEEE J. Sel. Top. Quantum Electron..

[86] Shoji T., Tsuchizawa T., Watanabe T., Yamada K., Morita H. (2002). Low loss mode size converter from 0.3 μm square Si wire waveguides to singlemode fibres. Electron. Lett..

[87] Core T., Tsang W., Sherman S. (1993). Fabrication Technology for an Integrated Surface-Micromachined Sensor. Solid State Technol..

[88] Chollet F., Liu H. A (not so) short introduction to Micro Electro Mechanical Systems, 5.2 ed. http://memscyclopedia.org/introMEMS.html.

[89] Guerre R., Hibert C., Burri Y., Flückiger P., Renaud P. (2005). Fabrication of vertical digital silicon optical micromirrors on suspended electrode for guided-wave optical switching applications. Sens. Actuators A Phys..

[90] Chollet F., Hegde G.M., Zhang X., Liu A., Asundi A. (2004). Vibration measurement with a micromachined mirror in a very-short external cavity laser. Sensors and Actuators A.

[91] Chollet F. SU-8: Thick Photo-Resist for MEMS. http://memscyclopedia.org/su8.html.

[92] Liu H., Chollet F. (2006). Micro Fork Hinge for MEMS Devices. J. Exp. Mech..

[93] Liu H., Chollet F. (2006). Layout Controlled One-Step Dry Etch and Release of MEMS Using Deep RIE on SOI Wafer. J. Microelectromech. Syst..

[94] Marcatili E.A.J. (1969). Bends in Optical Dielectric Guides. Bell Syst. Tech. J..

[95] Jiang F., Keating A., Martyniuk M., Pratap R., Faraone L., Dell J.M. (2013). Process Control of Cantilever Deflection for Sensor Application Based on Optical Waveguides. J. Microelectromech. Syst..

[96] Terui H., Kobayashi M. (1981). Total Reflection Optical Waveguide Switching Through Dielectric Chip Motion. Appl. Opt..

[97] Yariv A. (1973). Coupled-mode theory for guided-wave optics. J. Quant. Electron..

[98] Horak P., Stewart W., Loh W.H. Coupled waveguides with MEMS actuation for continuously tunable optical buffering. Proceedings of 2012 14th International Conference on Transparent Optical Networks (ICTON).

[99] Hah D., Bordelon J., Zhang D. (2011). Mechanically tunable optical filters with a microring resonator. Appl. Opt..

[100] Aksyuk V.A. (2015). Design and modeling of an ultra-compact 2x2 nanomechanical plasmonic switch. Opt. Express.

[101] Feynman R.P. (1992). There’s plenty of room at the bottom. J. Microelectromech. Syst..

